# Tracking Control for Asymmetric Underactuated Sea Vehicles in Slow Horizontal Movement

**DOI:** 10.3390/s25134205

**Published:** 2025-07-05

**Authors:** Przemyslaw Herman

**Affiliations:** Institute of Automatic Control and Robotics, Poznan University of Technology, ul. Piotrowo 3a, 60-965 Poznan, Poland; przemyslaw.herman@put.poznan.pl

**Keywords:** underactuated underwater vehicle, tracking control, backstepping, integral sliding mode control, robustness, simulation testing

## Abstract

In this paper, a robust tracking control problem for underactuated underwater vehicles in horizontal motion is investigated. The presented control scheme that performs the trajectory tracking task is a combination of the backstepping technique and the integral sliding mode control method using the inertial quasi velocities (IQVs) resulting from the inertia matrix decomposition. Unlike many known solutions, the proposed approach allows not only trajectory tracking, but also, due to the fact that IQV includes dynamic and geometric model parameters, allows us to obtain additional information about changes in vehicle behavior during movement. In this way, some insight into its dynamics is obtained. Moreover, the control strategy takes into account model inaccuracies and external disturbances, which makes it more useful from a technical point of view. Another advantage of this work is to indicate problems occurring during the implementation of trajectory tracking in algorithms with a dynamics model containing a diagonal inertia matrix, i.e., without inertial couplings. The theoretical results are illustrated by simulation tests conducted on two models of underwater vehicles with three degrees of freedom (DOF).

## 1. Introduction

There are many problems associated with underwater vehicles, including underactuation, speed limitations, and lumped dynamics due to parameter uncertainties and external disturbances. A very attractive task for the movement of various marine vehicles is tracking control, which allows the system to achieve the desired trajectory with satisfactory performance. Since this work only deals with the implementation of trajectory tracking in horizontal motion for underactuated vehicles, only a brief review of the literature on this class of vehicles is limited.

Very often, the dynamics model assumes full symmetry of the vehicle, which means that the center of mass coincides with the geometric center. Then, a control strategy is built for the adopted model. On the one hand, the advantage of this approach is that the mathematical theory is simplified. On the other hand, if the symmetry condition is not met in practice, the implementation of the control task will be difficult or even impossible. Despite this obvious observation, many proposals have been made to solve the trajectory tracking problem.

The existing control strategies are based on different control methods or their combinations. Some examples include the use of sliding mode control (SMC) [1], prescribed performance for trajectory tracking error [2], contraction theory [3], combination of backstepping and SMC [4], backstepping and integral SMC [5], an adaptive fixed-time terminal sliding mode control (AFiTSMC) [6], an adaptive trajectory tracking controller with a new integral-type Lyapunov based on disturbance observer [7], and model predictive control with SMC (theory and simulations supported by the experiment) [8]. In many works, in addition to other control algorithms, artificial neural networks (ANNs) are used to obtain better performance of the proposed trajectory tracking strategy, e.g., ref. [9] (with backstepping and SMC), ref. [10] (with event-triggered control (ETC) and backstepping), ref. [11] (with prescribed performance control (PPC), barrier Lyapunov function (BLF), and dynamic surface control (DSC)), ref. [12] (non-singular TSMC). Additionally, it is worth pointing out some works based on fuzzy logic, e.g., ref. [13] (with event-triggered control and dynamic surface control), ref. [14] (with adaptive backstepping technique, command-filter, and event-triggered mechanism). Sometimes, however, the values of the forces and moments for the vehicle selected for testing do not seem realistic, e.g., in [15,16], and the results obtained indicate that the algorithm is effective, but not necessarily for real operating conditions.

Even based on this brief literature review, it is easy to see that control strategies focus on the use of increasingly complex methods (or rather their combinations to precisely accomplish the trajectory tracking task). However, it is difficult to know whether acceptable results will also be obtained in the case of vehicle asymmetry. Herman [17] shows, based on simulation tests for several selected control algorithms, one vehicle model, and two trajectories, that strategies based on a fully symmetric model do not sufficiently perform the task of tracking the trajectory or are even unable to perform this task if there are shifts in the center of mass.

There are also works in which control schemes based on such a reduced model were verified experimentally. One can then somehow argue that with the adopted assumptions, the problems of lack of symmetry have been overcome. For example, ref. [18] (adaptive single-parameter backstepping controller (SPBC) with saturation) or [19] (the radial basis function and model reference adaptive control) may be mentioned here. It can be assumed that if the proposed control schemes were verified on a real vehicle, this work would not address this issue.

There is also a group of control strategies based on trajectory tracking using vehicle models for which the center of mass is shifted relative to the geometric center. Such models include mass couplings and hydrodynamic damping couplings. Taking this shift into account in the model brings it closer to reality than using a symmetrical vehicle model and makes the control scheme more useful. There are also some works that assume asymmetric vehicle models for control purposes. For example, the SMC method was given in [20]. A control scheme applying backstepping and integral SMC was shown and tested in [21]. The scheme developed in [22] was composed of the backstepping technique, cascade analysis, and the Lyapunov approach. In [23], a control strategy using the input–output feedback linearization method was proposed (with results obtained for a real vehicle), while in [24], output feedback control using a state observer was considered.

It is also worth mentioning the use of ANN in control strategies, e.g., ref. [25] (with prescribed performance and transverse function control), ref. [26] (optimal control with adaptive dynamic programming), and [27] (with prescribed trajectory tracking performance). Unfortunately, sometimes it happens that the performance of the control algorithms was verified by simulation, but the obtained input signal values were unrealistic, e.g., in [28].

There are few works in which experimental results obtained for real surface vehicles with an asymmetric model can be found, e.g., refs. [29,30]. Thus, it can be concluded that experimental validation is difficult in the case of a shift of the center of mass, and therefore, simulation studies may indicate directions for further research in the field of trajectory tracking. It is also worth noting that there is a very limited number of papers in which the control scheme has been tested for two vehicle models. This approach proves a certain universality of the trajectory tracking algorithm. One could mention here, e.g., refs. [21,31].

The following thesis of the work is stated: if it is not possible, for any reason, to experimentally verify the control scheme on a real vehicle, it should be tested in simulation, taking into account the shift of the center of mass.

It can be seen that there are many control schemes for a model with a diagonal inertia matrix (i.e., assuming that the geometric center of the vehicle is located in the same place as the geometric center). Therefore, it seems interesting to check the suitability of already designed and simulation-tested control schemes for tracking the trajectories in the event of an intentional or unintentional shift of the center of mass. This work is an attempt to consider this issue because with the constantly growing number of proposed control strategies, perhaps some of them are robust to the event of center of mass shift, while others are not suitable for this purpose and require almost ideal operating conditions, which in practice may turn out to be impossible.

The slow motion of the vehicle (below 1 m/s in steady state motion phase) was chosen for two reasons. Firstly, the effect of couplings can be demonstrated when the dynamic forces resulting from vehicle movement are still small compared to movement at the permissible velocity. Secondly, if the algorithms designed for the dynamics model without taking into account the effect resulting from the shift of the center of mass were tested at high velocities, then the errors could be unacceptable.

The novelty of this work is the proposal of a combination of the backstepping and SMC methods, but additionally based on the velocity transformation method, which results in obtaining variables containing dynamic and geometric parameters of the vehicle model. Such an approach allows for including inertial couplings in the dynamic model, which leads to estimating their influence on the implementation of the trajectory tracking task in the designed controller (taking into account IQV). It is equivalent to recognizing the effect of shifting the vehicle’s center of mass on its behavior when it is in motion. It is therefore a certain extension of known control methods to the case when the vehicle dynamics model is generalized. In many works known from the literature (as mentioned earlier), the dynamics model does not take into account the couplings in the system, and the controller is designed for such a model. This simplifies the mathematical form of the controller, but the situation when the center of mass is shifted is omitted. Sometimes, of course, a model with displacement of the center of mass is assumed, but in the longitudinal direction, which also does not provide full information about the performance of the control algorithm. This work shows that the problem resulting from the simplification of the model has a significant impact on the operation and performance of the closed-loop system. Another drawback that sometimes occurs is the lack of limits on the thrust forces, which can result in the control strategy not being able to be implemented in practice. Therefore, here the real limits on the drives for the tested marine vehicles are introduced. The offered control scheme is designed under the assumption that the dynamics model has a symmetric inertia matrix, which means that couplings resulting from the fact that the center of mass is not identically located as the geometric center are taken into account. Of course, when both points are in the same place, the scheme can also be used.

Compared with the results available in the literature, the main contributions of this work can be summarized as follows.

(1) A new form of control algorithm (for asymmetric vehicle model) based on the IQV for a thre DOF underactuated vehicle is developed. The algorithm enables tracking the position trajectory, and it is robust to not fully known dynamics and external disturbances when inertial couplings are taken into consideration.
(2) Unlike [32,33], inaccurate knowledge of the model parameters is taken into account in the control scheme. A model with three DOF is considered instead of five DOF, but assuming the presence of inertial couplings (which was not the case in the cited works). Moreover, the proposed control method differs from the algorithms described in [21,34] by using a different control concept.
(3) The controller can be used to gain additional insight into the vehicle dynamics model, e.g., information about the kinetic energy corresponding to each variable speed deformation caused by couplings between variables is obtained.
(4) In additional simulation tests (other than in [17]), two selected algorithms suitable for situations where there is no center of mass shift (for a symmetric vehicle model) are investigated to check how they perform the task of tracking the trajectory when there is a displacement of the center of mass. Two variants of moving the center of mass in the longitudinal and lateral direction are considered. Such simulation verification of the control strategies that assume no shift of the center of mass seems necessary if there is no experimental verification. It is worth noting that this type of algorithm for fully symmetric vehicles is proposed very often.

The rest of this paper is organized as follows. The mathematical model of the vehicle is shown in Section 2. Section 3 presents the equations of motion after velocity transformation, which include inertial quasi-velocities (IQVs). The proposed trajectory tracking controller is presented in Section 4. The numerical test conditions are discussed in Section 5. The simulation results of the performance of the proposed controller are given in Section 6. Section 7 describes the performance of two other selected control strategies. Section 8 gives additional analysis and discussion of the results. The conclusions of this paper and possible further research are offered in Section 9.

## 2. Mathematical Model of Vehicle Moving in Horizontal Plane

**Figure 1:**
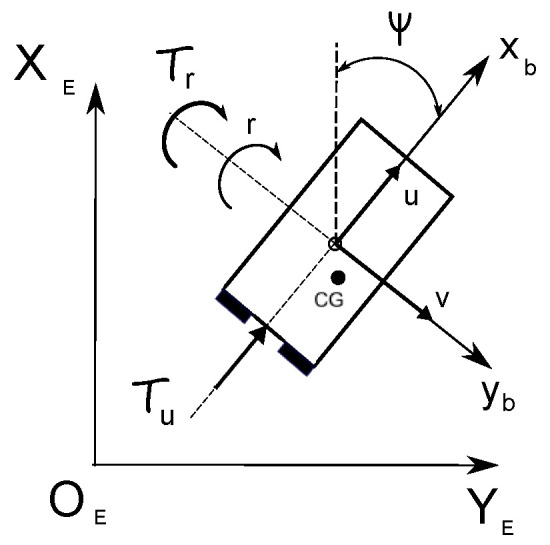
The underactuated vehicle coordinate system.

The underactuated marine vehicle under consideration given in Figure 1 can be described by [35]:

(1) $$\dot{\eta }=J\left(\psi \right)\nu ,$$

(2) $$M\dot{\nu }=-C\left(\nu \right)\nu -D\left(\nu \right)\nu +\tau +f_{d},$$

where $\eta ={\left[x,y,\psi \right]}^{T}$ denotes the position in the Earth-fixed frame and $\psi \in \left[0,2\pi \right)$ (or $\psi \in \left[0,-2\pi \right)$) is yaw angle, $\nu ={\left[u,v,r\right]}^{T}$ represents the velocity vector (surge, sway, and yaw velocities in the body-fixed frame), J(ψ) is the transformation matrix, *M* means the vehicle inertia matrix, C(ν) means the Coriolis and centripetal matrix, and D(ν) means the matrix of hydrodynamic damping. The input control vector $\tau ={\left[\tau_{u},0,\tau_{r}\right]}^{T}$ contains the thrust force $\tau_{u}$ and the yaw torque $\tau_{r}$. The vector of time-varying disturbances is denoted as $f_{d}$.

The used matrices and vectors are described by:

(3) $$\begin{array}{l} J\left(\psi \right)=\left[\begin{array}{ccc} \cos\psi & -\sin\psi & 0\\ \sin\psi & \cos\psi & 0\\ 0 & 0 & 1 \end{array}\right],\;M=\;\left[\begin{array}{ccc} m_{11} & \;0\; & m_{13}\\ 0 & \;m_{22}\; & m_{23}\\ m_{13} & \;m_{23}\; & m_{33} \end{array}\right]\;,\\ C\left(\nu \right)=\;\left[\begin{array}{ccc} 0 & \;0\; & c_{13}\\ 0 & \;0\; & c_{23}\\ -c_{13} & \;-c_{23}\; & 0 \end{array}\right],\;D\left(\nu \right)=\;\left[\begin{array}{ccc} d_{11} & \;0\; & d_{13}\\ 0 & \;d_{22}\; & d_{23}\\ d_{31} & \;d_{32}\; & d_{33} \end{array}\right],\;f_{d}=\;\left[\begin{array}{c} f_{du}\\ f_{dv}\\ f_{dr} \end{array}\right]. \end{array}$$

The left-handed coordinate system was adopted to ensure consistency with J(ψ). Elements of the matrices *M*, C(ν), and D(ν) are defined in Appendix A.

**Assumption 1.**  *The origin of the body-fixed frame is located in the geometric center of the vehicle.*

**Assumption 2.**  *The desired reference trajectory $\eta_{d}={\left[x_{d},y_{d}\right]}^{T}$ of the system, and its first-order differential ${\dot{\eta }}_{d}={\left[{\dot{x}}_{d},{\dot{y}}_{d}\right]}^{T}$, are regular, smooth, bounded, and available.*

**Assumption 3.**  *The model’s inaccuracies (3) are limited, which means that its parameter perturbations are bounded as in [36,37], i.e., $|m_{ij}-{\hat{m}}_{ij}|\leq {\bar{m}}_{ij}$, $|d_{ij}-{\hat{d}}_{ij}|\leq {\bar{d}}_{ij}$, where i,j=1,2,3 (hat symbol denotes the nominal value of the real parameter).*

**Assumption 4.**  *The external disturbances are bounded and time-varying, namely: $\left|\right|f_{du}\left|\right|\leq w_{1},\;\;||f_{dv}\left|\right|\leq w_{2},\;\;||f_{dr}\left|\right|\leq w_{3}$, where $w_{1},w_{2},w_{3}$ are constants representing the appropriate unknown bounds.*

**Assumption 5.**  *The velocities u,v,r and control signals $\tau_{u},\tau_{r}$ of the vehicle are bounded, i.e., $u_{min}\leq u\leq u_{max}$, $v_{min}\leq vs.\leq v_{max}$, $r_{min}\leq r\leq r_{max}$, $\tau_{u\;min}\leq \tau_{u}\leq \tau_{u\;max}$, and $\tau_{r\;min}\leq \tau_{r}\leq \tau_{r\;max}$ [38,39]. It arises from practical engineering [37,39]. The assumption of thrust saturation is acceptable when the effect is not severe, meaning that control input values exceed the thruster limit only occasionally [40].*

## 3. Inertial Quasi-Velocities Based Equations

In order to decompose the inertial matrix *M* and to obtain the dynamics equation containing IQV, the inertial matrix must be symmetric. There are various decomposition methods, but the method from [41] is used here as it has been successfully implemented for marine vehicles, e.g., in [21,34].

**Remark 1.**  *Decomposing the matrix M, one has a positive definite diagonal matrix $N={\hat{\Phi }}^{T}M\hat{\Phi }$ (it has the properties of the M matrix). So this means that $M={\hat{\Phi }}^{-T}N{\hat{\Phi }}^{-1}$. The $\hat{\Phi }$ matrix contains nominal parameters, while any inaccuracies of* Φ *are shifted to the vector $\tau_{d}=f_{d}+f\left(\Delta \Phi \right)$ defined as $\tau_{d}={\left[\tau_{du},\tau_{dv},\tau_{dr}\right]}^{T}$. However, the decomposition of the matrix $\hat{M}$ into nominal parameters leads to the matrix $\hat{N}={\hat{\Phi }}^{T}\hat{M}\hat{\Phi }$. Therefore, the term f(ΔΦ) represents the inertial forces resulting from the use of two different matrices N and $\hat{N}$.*

The following additional assumptions are valid.

**Assumption 6.**  *Taking into account Assumption 3, $|N_{i}-{\hat{N}}_{i}|\leq {\bar{N}}_{i}$, where i=1,2,3 also applies.*

**Assumption 7.**  *Due to the fact that parameter disturbances are taken into account in the control scheme (this is the essence of the method), it is allowed to use the vector $f_{d}$ instead of the vector $\tau_{d}$, that is $f_{d}=\tau_{d}$.*

**Assumption 8.**  *Recalling* Assumption 4 *is also $\left|\right|f_{d\zeta_{3}}\left|\right|={\hat{\Phi }}_{13}\left|\right|f_{du}\left|\right|+{\hat{\Phi }}_{23}\left|\right|f_{dv}\left|\right|+\left|\right|f_{dr}\left|\right|\leq w_{2c}$, where $w_{2c}$ is a constants concerning an unknown bound.*

With these assumptions, the new equations, instead of (2), have the form:

(4) $$N\dot{\zeta }=-\left({\hat{\Phi }}^{T}C\left(\nu \right)\nu +{\hat{\Phi }}^{T}D\left(\nu \right)\nu \right)+{\hat{\Phi }}^{T}\tau +{\hat{\Phi }}^{T}f_{d},$$

(5) $$\begin{array}{c} \nu =\hat{\Phi }\;\zeta ,\\ \hat{\Phi }=\left[\begin{array}{ccc} 1 & 0 & {\hat{\Phi }}_{13}\\ 0 & 1 & {\hat{\Phi }}_{23}\\ 0 & 0 & 1 \end{array}\right],\;N=diag\left\{N_{1},N_{2},N_{3}\right\}. \end{array}$$

Considering the elements defined in Appendix A, the vector of the inertial quasi-velocities can be written as $\zeta ={\left[\zeta_{1},\zeta_{2},\zeta_{3}\right]}^{T}$. The quantities $N_{1}=m_{11}$, $N_{2}=m_{22}$, $N_{3}=m_{33}-\left(m_{13}^{2}/m_{11}\right)-\left(m_{23}^{2}/m_{22}\right)$, ${\hat{\Phi }}_{13}=-\left({\hat{m}}_{13}/{\hat{m}}_{11}\right)$, ${\hat{\Phi }}_{23}=-\left({\hat{m}}_{23}/{\hat{m}}_{22}\right)$ result from the decomposition of the matrix *M*.

New equations of motion replacing (4) and (5) are as follows:

(6) $$\zeta_{1}=u-{\hat{\Phi }}_{13}r,$$

(7) $$\zeta_{2}=v-{\hat{\Phi }}_{23}r,$$

(8) $$\zeta_{3}=r,$$

(9) $$N_{1}{\dot{\zeta }}_{1}=H_{1}\left(\zeta \right)+\tau_{u}+f_{du},$$

(10) $$N_{2}{\dot{\zeta }}_{2}=H_{2}\left(\zeta \right)+f_{dv},$$

(11) $$N_{3}{\dot{\zeta }}_{3}=H_{3}\left(\zeta \right)+\tau_{\zeta_{3}}+f_{d\zeta_{3}},$$

with $\tau_{\zeta_{3}}={\hat{\Phi }}_{13}\tau_{u}+\tau_{r}$, $\tau_{d\zeta_{3}}={\hat{\Phi }}_{13}f_{du}+{\hat{\Phi }}_{23}f_{dv}+f_{dr}$ and:

(12) $$H_{1}\left(\zeta \right)=\left(m_{22}v+m_{23}r\right)r-d_{11}u-d_{13}r,$$

(13) $$H_{2}\left(\zeta \right)=\left(-m_{11}u+m_{13}r\right)r-d_{22}v-d_{23}r,$$

(14) $$\begin{array}{cc} H_{3}\left(\zeta \right)= & -\left(m_{22}v+m_{23}r\right)u+\left(m_{11}u-m_{13}r\right)vs.+{\hat{\Phi }}_{13}\left(m_{22}v+m_{23}r\right)r-{\hat{\Phi }}_{23}\left(m_{11}u-m_{13}r\right)r\\ & -\left({\hat{\Phi }}_{13}d_{11}+d_{31}\right)u-\left({\hat{\Phi }}_{23}d_{22}+d_{32}\right)v-\left({\hat{\Phi }}_{13}d_{13}+{\hat{\Phi }}_{23}d_{23}+d_{33}\right)r. \end{array}$$

For simplicity, the symbols $H_{1}=H_{1}\left(\zeta \right)$, $H_{2}=H_{2}\left(\zeta \right)$, and $H_{3}=H_{3}\left(\zeta \right)$ are introduced.

## 4. Trajectory Tracking Controller Design

In this section, the desired trajectory tracking control algorithm, including IQV, will be designed. The control scheme is inspired by the results of [33] (this concept was also developed in [32]). However, in the mentioned work, as well as in [32], only a model with a diagonal inertia matrix was considered.

When an asymmetric vehicle model is taken into account, the inertia matrix is symmetrical, and solving the task of tracking the desired trajectory is more difficult and not so obvious. Moreover, the proposed algorithm takes into account the dynamic components of the vehicle model in the form representing the percentage of inaccurate knowledge of the parameters of this model.

Using (9) and (11), the accelerations $\dot{u}$ and $\dot{r}$ can be obtained in the form:

(15) $$\dot{u}=a_{u}+b_{u}\tau_{u}+b_{ur}\tau_{r},$$

(16) $$\dot{r}=a_{r}+b_{r}\tau_{r}+b_{ur}\tau_{u},$$

where:

(17) $$a_{u}=N_{1}^{-1}\left(H_{1}+\tau_{du}\right)+{\hat{\Phi }}_{13}N_{3}^{-1}\left(H_{3}+\tau_{d\zeta_{3}}\right),\;\;b_{u}=N_{1}^{-1}+{\hat{\Phi }}_{13}^{2}N_{3}^{-1},$$

(18) $$b_{ur}={\hat{\Phi }}_{13}N_{3}^{-1},\;\;a_{r}=N_{3}^{-1}\left(H_{3}+\tau_{d\zeta_{3}}\right),\;\;b_{r}=N_{3}^{-1}.$$

Since the objective of the algorithm is to track a desired trajectory $x_{d},y_{d}$, then two errors of the form are defined:

(19) $$e_{x}=x-x_{d},\;\;e_{y}=y-y_{d}.$$

The calculation of the second derivative of these position errors allows the use of available control signals $\tau_{u},\tau_{r}$ from (15) and (16), applying $\dot{\psi }=r$:

(20) $$\begin{array}{cc} {\ddot{e}}_{x} & =\dot{u}\cos\psi -u\dot{\psi }\sin\psi -\dot{v}\sin\psi -vs.\dot{\psi }\cos\psi -{\ddot{x}}_{d}\\ & =\left(a_{u}+b_{u}\tau_{u}+b_{ur}\tau_{r}\right)\cos\psi -ur\sin\psi -\dot{v}\sin\psi -vs.r\cos\psi -{\ddot{x}}_{d}, \end{array}$$

(21) $$\begin{array}{cc} {\ddot{e}}_{y} & =\dot{u}\sin\psi +u\dot{\psi }\cos\psi +\dot{v}\cos\psi -vs.\dot{\psi }\sin\psi -{\ddot{y}}_{d}\\ & =\left(a_{u}+b_{u}\tau_{u}+b_{ur}\tau_{r}\right)\sin\psi +ur\cos\psi +\dot{v}\cos\psi -vs.r\sin\psi -{\ddot{y}}_{d}. \end{array}$$

Denoting now:

(22) $$\begin{array}{c} \;Y_{1}=a_{u}\cos\psi -ur\sin\psi -\dot{v}\sin\psi -vs.r\cos\psi -{\ddot{x}}_{d},\\ Y_{2}=a_{u}\sin\psi +ur\cos\psi +\dot{v}\cos\psi -vs.r\sin\psi -{\ddot{y}}_{d}, \end{array}$$

it can be written in the matrix form $e=Y+\Gamma \tau_{A}$, i.e.,

(23) $$\left[\begin{array}{c} {\ddot{e}}_{x}\\ {\ddot{e}}_{y} \end{array}\right]=\left[\begin{array}{c} Y_{1}\\ Y_{2} \end{array}\right]+\left[\begin{array}{cc} b_{u}\cos\psi & b_{ur}\cos\psi \\ b_{u}\sin\psi & b_{ur}\sin\psi \end{array}\right]\left[\begin{array}{c} \tau_{u}\\ \tau_{r} \end{array}\right],$$

where $Y={\left[Y_{1},Y_{2}\right]}^{T}$, and $\tau_{A}={\left[\tau_{u},\tau_{r}\right]}^{T}$ which represents the actuated input signals. However, from (23), it can be seen that the matrix Γ is singular because its determinant is equal to zero. Therefore, it is not possible to directly control with $\tau_{u}$ and $\tau_{r}$. Instead, kinematic relationships can be used as in [42] and later in [33].

Defining:

(24) $${\dot{\eta }}_{1}=J_{1}\left(\psi \right)\nu_{1},$$

(25) $$\nu_{1}=\left[\begin{array}{c} u\\ vs. \end{array}\right],\;\;{\dot{\eta }}_{1}=\left[\begin{array}{c} \dot{x}\\ \dot{y} \end{array}\right],\;\;J_{1}\left(\psi \right)=\left[\begin{array}{cc} \cos\psi & -\sin\psi \\ \sin\psi & \cos\psi \end{array}\right],\;\;S\left(r\right)=\left[\begin{array}{cc} 0 & -r\\ r & 0 \end{array}\right],$$

one has $S\left(r\right)=-S^{T}\left(r\right)$ and the time derivative of the function $J_{1}\left(\psi \right)\equiv J_{1}$ can be determined in the form:

(26) $${\dot{J}}_{1}=J_{1}S\left(r\right).$$

Assuming that the actual position is $\eta_{1}={\left[x,y\right]}^{T}$ and the desired position is $\eta_{1d}={\left[x_{d},y_{d}\right]}^{T}$, one obtains the tracking position error ${\left[e_{x},e_{y}\right]}^{T}=\eta_{1}-\eta_{1d}$. Now, the new position error is defined as follows:

(27) $$\sigma =v_{p}-\Delta ,\;\;v_{p}={\left[v_{p1},v_{p2}\right]}^{T},\;\;v_{p}=J_{1}^{-1}\left(\eta_{1}-\eta_{1d}\right),\;\;\Delta ={\left[\delta ,0\right]}^{T},$$

where $v_{p}$ means the position error in the body-fixed frame and Δ is the constant error margin. Taking the above quantities into account, one can calculate the derivative of the variable σ:

(28) $$\begin{array}{cc} \dot{\sigma } & ={\dot{v}}_{p}-\dot{\Delta }={\dot{J}}_{1}^{-1}\left(\eta_{1}-\eta_{1d}\right)+J_{1}^{-1}\left({\dot{\eta }}_{1}-{\dot{\eta }}_{1d}\right)=-S\left(r\right)\sigma -S\left(r\right)\Delta +\nu_{1}-{\dot{J}}_{1}^{-1}\eta_{1d}\\ & =-S\left(r\right)\sigma -\left[\begin{array}{cc} 0 & -r\\ r & 0 \end{array}\right]\left[\begin{array}{c} \delta \\ 0 \end{array}\right]+\left[\begin{array}{c} u\\ vs. \end{array}\right]-\left[\begin{array}{c} 0\\ r\delta \end{array}\right]+\left[\begin{array}{c} u\\ 0 \end{array}\right]+\left[\begin{array}{c} 0\\ vs. \end{array}\right]-{\dot{J}}_{1}^{-1}\eta_{1d}. \end{array}$$

Consequently, one obtains:

(29) $$\dot{\sigma }=-S\left(r\right)\sigma +\left[\begin{array}{cc} 1 & 0\\ 0 & -\delta \end{array}\right]\left[\begin{array}{c} u\\ r \end{array}\right]+\left[\begin{array}{c} 0\\ vs. \end{array}\right]-{\dot{J}}_{1}^{-1}\eta_{1d}.$$

However, due to the fact that the controller should include quasi-velocities (6) and (8), the following relationship should be used instead $u_{e}=u-u_{d}$, $r_{e}=r-r_{d}$:

(30) $$\zeta_{1e}=\zeta_{1}-\zeta_{1d}=u-u_{d}-{\hat{\Phi }}_{13}\left(r-r_{d}\right)=u_{e}-{\hat{\Phi }}_{13}r_{e},\;\;\zeta_{3e}=r_{e}.$$

Considering the above equation, the second component on the right will be of the form:

(31) $$\left[\begin{array}{cc} 1 & 0\\ 0 & -\delta \end{array}\right]\left(\left[\begin{array}{c} \zeta_{1e}+{\hat{\Phi }}_{13}\zeta_{3e}\\ \zeta_{3e} \end{array}\right]+\left[\begin{array}{c} \zeta_{1d}+{\hat{\Phi }}_{13}\zeta_{3d}\\ \zeta_{3d} \end{array}\right]\right)\;\;\;\;\;\;\;\;\;\;\;\;\;=\left[\begin{array}{cc} 1 & {\hat{\Phi }}_{13}\\ 0 & -\delta \end{array}\right]\left(\left[\begin{array}{c} \zeta_{1e}\\ \zeta_{3e} \end{array}\right]+\left[\begin{array}{c} \zeta_{1d}\\ \zeta_{3d} \end{array}\right]\right).$$

Therefore, (29) can be written as follows:

(32) $$\dot{\sigma }=-S\left(r\right)\sigma +\Pi \left(\left[\begin{array}{c} \zeta_{1e}\\ \zeta_{3e} \end{array}\right]+\left[\begin{array}{c} \zeta_{1d}\\ \zeta_{3d} \end{array}\right]\right)+\left[\begin{array}{c} 0\\ vs. \end{array}\right]-{\dot{J}}_{1}^{-1}\eta_{1d},\;\;\mathrm{where}\;\;\Pi =\left[\begin{array}{cc} 1 & {\hat{\Phi }}_{13}\\ 0 & -\delta \end{array}\right].$$

The desired quasi-velocities are determined from the relationship:

(33) $$\left[\begin{array}{c} \zeta_{1d}\\ \zeta_{3d} \end{array}\right]=\Pi^{-1}\left(-K\sigma -\left[\begin{array}{c} 0\\ vs. \end{array}\right]+{\dot{J}}_{1}^{-1}\eta_{1d}\right),$$

where K=k·I, k>0, $I\in R^{2\times 2}$. Inserting (33) into (32), one obtains:

(34) $$\dot{\sigma }=-\left(S\left(r\right)+K\right)\sigma +\Pi \left[\begin{array}{c} \zeta_{1e}\\ \zeta_{3e} \end{array}\right].$$

The trajectory tracking according the relationship (27) can be applied if (1) the matrix $\dot{\sigma }=-\left(S\left(r\right)+K\right)\sigma$ is uniformly exponential stable, and (2) ${\left[\zeta_{1e},\zeta_{3e}\right]}^{T}$ is uniformly exponentially convergent to zero. Consider then the following equation:

(35) $$\dot{X}\left(t\right)=A\left(t\right)X\left(t\right),\;\;X\left(t_{0}\right)=X_{0},\;\;A\in R^{n\times n}$$

In the paper [42] (Theorem 1, Corollary 1), it is proved that condition (1) is satisfied for k>0. In order to satisfy condition (2), that is, to ensure that the signal vector ${\left[\zeta_{1e},\zeta_{3e}\right]}^{T}$ converges to zero, the SMC method was used. The sliding surfaces were proposed as follows:

(36) $$S_{1}=g_{1}\int_{0}^{t}\zeta_{1e}\left(\iota \right)d\iota +\zeta_{1e},$$

(37) $$S_{2}=g_{2}\int_{0}^{t}\zeta_{3e}\left(\iota \right)d\iota +\zeta_{3e},$$

where $g_{1},g_{2}$ are some constants. Calculating the time derivative of $S_{1},S_{2}$ and recalling Assumption 7, one obtains:

(38) $$\begin{array}{cc} {\dot{S}}_{1} & =g_{1}\zeta_{1e}+{\dot{\zeta }}_{1e}=g_{1}\zeta_{1e}+N_{1}^{-1}\left(H_{1}+\tau_{u}+f_{du}\right)-{\dot{\zeta }}_{1d}\\ & =N_{1}^{-1}\left(H_{1}+\tau_{u}+f_{du}-N_{1}{\dot{\zeta }}_{1d}+N_{1}g_{1}\zeta_{1e}\right), \end{array}$$

(39) $$\begin{array}{cc} {\dot{S}}_{2} & =g_{2}\zeta_{3e}+{\dot{\zeta }}_{3e}=g_{2}\zeta_{3e}+N_{3}^{-1}\left(H_{3}+\tau_{\zeta_{3}}+f_{d\zeta_{3}}\right)-{\dot{\zeta }}_{3d}\\ & =N_{3}^{-1}\left(H_{3}+\tau_{\zeta_{3}}+f_{d\zeta_{3}}-N_{3}{\dot{\zeta }}_{3d}+N_{3}g_{2}\zeta_{3e}\right). \end{array}$$

Next, the sliding mode control vector is introduced $\tau =\tau_{D}+\tau_{S}$, where $\tau ={\left[\tau_{u},\tau_{\zeta_{3}}\right]}^{T}$ ($\tau_{u}=\tau_{\zeta_{1}}$). It consists of two components, i.e., first $\tau_{D}={\left[\tau_{D1},\tau_{D3}\right]}^{T}$ enables dynamics compensation whereas the second $\tau_{S}={\left[\tau_{S1},\tau_{S3}\right]}^{T}$ allows to reach the sliding surface rate:

(40) $$\tau_{D1}=-{\hat{H}}_{1}+{\hat{N}}_{1}{\dot{\zeta }}_{1d}-{\hat{N}}_{1}g_{1}\zeta_{1e},\;\;\;\tau_{S1}=-\beta_{1}\tanh\left(S_{1}\right),$$

(41) $$\tau_{D2}=-{\hat{H}}_{3}+{\hat{N}}_{3}{\dot{\zeta }}_{3d}-{\hat{N}}_{3}g_{2}\zeta_{1e},\;\;\;\tau_{S1}=-\beta_{2}\tanh\left(S_{2}\right).$$

The control signals can be applied in case of accurate knowledge of the model parameters. However, here, the model is not known exactly and, in addition, there are external disturbances (Assumption 4).

If the dynamic model is not known exactly, then instead of (38) and (39), one obtains:

(42) $${\dot{S}}_{1}=N_{1}^{-1}\left(H_{1}-{\hat{H}}_{1}+\left({\hat{N}}_{1}-N_{1}\right){\dot{\zeta }}_{1d}+\left(N_{1}-{\hat{N}}_{1}\right)g_{1}\zeta_{1e}+f_{du}+\tau_{u}\right),$$

(43) $${\dot{S}}_{2}=N_{3}^{-1}(H_{3}-{\hat{H}}_{3}+\left({\hat{N}}_{3}-N_{3}\right){\dot{\zeta }}_{3d}+\left(N_{3}-{\hat{N}}_{3}\right)g_{2}\zeta_{3e}+f_{d\zeta_{3}}+\tau_{\zeta_{3}}).$$

For the model under consideration, one obtains:

(44) $$H_{1}-{\hat{H}}_{1}=\left(m_{22}-{\hat{m}}_{22}\right)vr+\left(m_{23}-{\hat{m}}_{23}\right)r^{2}+\left({\hat{d}}_{11}-d_{11}\right)u+\left({\hat{d}}_{13}-d_{13}\right)r,$$

(45) $$\begin{array}{cc} H_{3}- & {\hat{H}}_{3}=\left({\hat{m}}_{22}-m_{22}\right)uv+\left(m_{11}-{\hat{m}}_{11}\right)uvs.+\left({\hat{m}}_{23}-m_{23}\right)ur+\left({\hat{m}}_{13}-m_{13}\right)vr\\ & +{\hat{\Phi }}_{13}\left(m_{22}-{\hat{m}}_{22}\right)vr+{\hat{\Phi }}_{13}\left(m_{23}-{\hat{m}}_{23}\right)r^{2}+{\hat{\Phi }}_{23}\left({\hat{m}}_{11}-m_{11}\right)ur+{\hat{\Phi }}_{23}\left(m_{13}-{\hat{m}}_{13}\right)r^{2}\\ & +\left({\hat{\Phi }}_{13}\left({\hat{d}}_{11}-d_{11}\right)+\left({\hat{d}}_{31}-d_{31}\right)\right)u+\left({\hat{\Phi }}_{23}\left({\hat{d}}_{22}-d_{22}\right)+\left({\hat{d}}_{32}-d_{32}\right)\right)vs.\\ & +\left({\hat{\Phi }}_{13}\left({\hat{d}}_{13}-d_{13}\right)+{\hat{\Phi }}_{23}\left({\hat{d}}_{23}-d_{23}\right)+\left({\hat{d}}_{33}-d_{33}\right)\right)r. \end{array}$$

The estimates of these values can be determined from:

(46) $${\dot{\hat{w}}}_{1}=\frac{1}{\mu_{1}}|S_{1}|,\;\;{\dot{\hat{w}}}_{2c}=\frac{1}{\mu_{2}}\left|S_{2}\right|,$$

where $\mu_{1},\mu_{2}$ mean some adaptive gains. Thus, the adaptive errors can be defined as:

(47) $${\tilde{w}}_{1}={\hat{w}}_{1}-w_{1},\;\;{\tilde{w}}_{2c}={\hat{w}}_{2c}-w_{2c}.$$

Taking into account the above considerations, the control signals should be modified to the form:

(48) $$\tau_{u}=-{\hat{H}}_{1}+{\hat{N}}_{1}{\dot{\zeta }}_{1d}-{\hat{N}}_{1}g_{1}\zeta_{1e}-\left(\Lambda_{1}+{\hat{w}}_{1}+\beta_{1}\right)\tanh\left(S_{1}\right),$$

(49) $$\tau_{\zeta_{3}}=-{\hat{H}}_{3}+{\hat{N}}_{3}{\dot{\zeta }}_{3d}-{\hat{N}}_{3}g_{2}\zeta_{1e}-\left(\Lambda_{2}+{\hat{w}}_{2}+\beta_{2}\right)\tanh\left(S_{2}\right),$$

where the gain functions $\Lambda_{1},\Lambda_{2}$ are:

(50) $$\Lambda_{1}={\bar{m}}_{22}\left|vr\right|+{\bar{m}}_{23}|r^{2}|+{\bar{d}}_{11}\left|u\right|+{\bar{d}}_{13}\left|r\right|+{\bar{N}}_{1}|{\dot{\zeta }}_{1d}|+g_{1}{\bar{N}}_{1}\left|\zeta_{1e}\right|+\rho_{1},$$

(51) $$\begin{array}{cc} \Lambda_{2}= & \left({\bar{m}}_{22}-{\bar{m}}_{11}\right)\left|uv\right|+\left({\bar{m}}_{13}+{\hat{\Phi }}_{13}{\bar{m}}_{22}\right)\left|vr\right|+\left({\bar{m}}_{23}+{\hat{\Phi }}_{23}{\bar{m}}_{11}\right)\left|ur\right|\\ & +\left({\hat{\Phi }}_{23}{\bar{m}}_{13}+{\hat{\Phi }}_{13}{\bar{m}}_{23}\right)|r^{2}|+\left({\bar{d}}_{31}+{\hat{\Phi }}_{13}{\bar{d}}_{11}\right)\left|u\right|+\left({\bar{d}}_{32}+{\hat{\Phi }}_{23}{\bar{d}}_{22}\right)\left|v\right|\\ & +\left({\bar{d}}_{33}+{\hat{\Phi }}_{13}{\bar{d}}_{13}+{\hat{\Phi }}_{23}{\bar{d}}_{23}\right)\left|r\right|+{\bar{N}}_{3}|{\dot{\zeta }}_{3d}|+g_{2}{\bar{N}}_{3}\left|\zeta_{3e}\right|+\rho_{2}. \end{array}$$

The constants $\rho_{1},\rho_{2}$ must be selected in the process of designing the controller.

**Theorem 1.**  *For the vehicle described by (1) and (4)–(14), if Assumptions 1 ÷ 8 are fulfilled, the vector ${\left[\zeta_{1e},\zeta_{3e}\right]}^{T}$ uniformly converges to neighborhood of zero.*

**Proof of Theorem 1.**  The Lyapunov function candidate is assumed in the following form:

(52) $$V=V_{1}+V_{2},\;\;\mathrm{with}\;\;V_{1}=\frac{1}{2}N_{1}S_{1}^{2}+\frac{1}{2}\mu_{1}{\tilde{w}}_{1}^{2},\;\;\;V_{2}=\frac{1}{2}N_{3}S_{2}^{2}+\frac{1}{2}\mu_{2}{\tilde{w}}_{2c}^{2}.$$

Calculating the time derivatives of the functions $V_{1}$ and $V_{2}$ and using (42)–(51) leads to the following:

(53) $$\begin{array}{cc} {\dot{V}}_{1} & =N_{1}S_{1}{\dot{S}}_{1}+\mu_{1}{\tilde{w}}_{1}{\dot{\tilde{w}}}_{1}=S_{1}(H_{1}-{\hat{H}}_{1}+\left({\hat{N}}_{1}-N_{1}\right){\dot{\zeta }}_{1d}+\left(N_{1}-{\hat{N}}_{1}\right)g_{1}\zeta_{1e}+\tau_{du}\\ & -\left(\Lambda_{1}+{\hat{w}}_{1}+\beta_{1}\right)\tanh\left(S_{1}\right))+\mu_{1}\left({\hat{w}}_{1}-w_{1}\right){\dot{\tilde{w}}}_{1}\leq S_{1}\left(\tau_{du}-\left(\rho_{1}+{\hat{w}}_{1}+\beta_{1}\right)\tanh\left(S_{1}\right)\right)\\ & +\left({\hat{w}}_{1}-w_{1}\right)|S_{1}|\leq -\left(\rho_{1}+\beta_{1}\right)\left|S_{1}\right|+\varsigma_{1}, \end{array}$$

(54) $$\begin{array}{cc} {\dot{V}}_{2} & =N_{3}S_{2}{\dot{S}}_{2}+\mu_{2}{\tilde{w}}_{2c}{\dot{\tilde{w}}}_{2c}=S_{2}(H_{3}-{\hat{H}}_{3}+\left({\hat{N}}_{3}-N_{3}\right){\dot{\zeta }}_{3d}+\left(N_{3}-{\hat{N}}_{3}\right)g_{2}\zeta_{3e}+\tau_{d\zeta_{3}}\\ & -\left(\Lambda_{2}+{\hat{w}}_{2c}+\beta_{2}\right)\tanh\left(S_{2}\right))+\mu_{2}\left({\hat{w}}_{2c}-w_{2c}\right){\dot{\tilde{w}}}_{2c}\leq S_{2}\left(\tau_{d\zeta_{3}}-\left(\rho_{2}+{\hat{w}}_{2c}+\beta_{2}\right)\tanh\left(S_{2}\right)\right)\\ & +\left({\hat{w}}_{2c}-w_{2c}\right)|S_{2}|\leq -\left(\rho_{2}+\beta_{2}\right)\left|S_{2}\right|+\varsigma_{2}, \end{array}$$

where $\varsigma_{1},\varsigma_{2}$ are some constants arising from the approximation of the model. From the above calculations, it follows that the sum of the derivatives $\dot{V}={\dot{V}}_{1}+{\dot{V}}_{2}$. Assuming $\varsigma =\varsigma_{1}+\varsigma_{2}$, one can write:

(55) $$\dot{V}\leq -\left(\rho_{1}+\beta_{1}\right)|S_{1}|-\left(\rho_{2}+\beta_{2}\right)\left|S_{2}\right|+\varsigma ,$$

which means that vector ${\left[\zeta_{1e},\zeta_{3e}\right]}^{T}$ is uniformly bounded. Considering the fulfillment of conditions (1) and (2), it can be concluded that the proposed scheme provides tracking of the desired trajectory.    □

**Remark 2.**  *From the fact that $\zeta_{1e}\to 0$, it follows that $u_{e}\to 0$ and $r_{e}\to 0$. If $\zeta_{3e}\to 0$, then $r_{e}\to 0$. Therefore, both variables result in $r_{e}\to 0$. Moreover, $\tau_{r}=\tau_{\zeta_{3}}-{\hat{\Phi }}_{13}\tau_{u}$.*

This result means that all tracking errors in the closed system converge to a small neighborhood of zero, and are therefore uniformly ultimately bounded. Taken together, the results conclude the proof.

Control signal calculation algorithm.

1. Calculation of accelerations $\dot{u},\dot{r}$ from (15) and (16).
2. Defining the position errors $e_{x},e_{y}$ from (19) and determination of their second derivative ${\ddot{e}}_{x},{\ddot{e}}_{y}$ with respect to time from (20) and (21), and next the vector (23).
3. Defining the new position error σ from (27), calculation of $\dot{\sigma }$ from (32), the desired quasi-velocities vector from (33), and next inserting the results into (34).
4. Defining the sliding surfaces $S_{1},S_{2}$ (36) and (37), and calculation of their time derivatives ${\dot{S}}_{1},{\dot{S}}_{2}$ from (38) and (39) for exactly known model or from (42) and (43) if the model contains inaccuracies.
5. Defining $\tau_{D1},\tau_{S1}$ from (40) and $\tau_{D2},\tau_{S2}$ from (41), and determination of their components from (44) and (45).
6. Calculation of control signals $\tau_{u},\tau_{\zeta_{3}}$ from (48) and (49) using $\Lambda_{1},\Lambda_{2}$ from (50) and (51).

## 5. Conditions for Numerical Tests

### 5.1. Models and Limitations

Three problems are considered in simulation studies, namely the following:

1. Test on two underwater vehicle models;
2. Limitation thrust forces;
3. Shifting the center of mass (asymmetry of the vehicle model).

Examination of the control scheme for two models should answer the question of whether the control algorithm can be used for vehicles with different dynamics. The vehicles taken for testing are applied in real marine research.

Limiting the thrust force causes the algorithm’s operating conditions to be close to real ones (of course, bearing in mind that these are simulation studies). In some works known from the literature, this condition is not met and therefore only information about the correct operation of the controller can be obtained, but without reference to real operating conditions.

Shifting the center of mass makes control difficult, so this issue is considered here. The inertia matrix is then symmetric. The proposed algorithm, from a theoretical point of view, is suitable to achieve this goal. Nevertheless, two other schemes for the dynamics model with a diagonal inertia matrix were tested in simulation studies. This is intended to answer the question whether such control strategies can be useful under the assumed operating conditions.

### 5.2. C-Ranger Vehicle

The C-Ranger AUV underwater vehicle was selected for testing. Its parameters, shown in Table 1, are taken from [43,44]. Because the matrix *M* should contain off-diagonal elements, then it is assumed that $m_{13}=m_{31}=12$ kg·m and $m_{23}=m_{32}=24$ kg·m (in source works, these values are absent, but they are needed for this test). These data correspond to $x_{g}=0.08$ m, $y_{g}=-0.03$ m, and $Y_{\dot{r}}=-7.5$ kg·m, $X_{\dot{r}}=-5.8$ kg·m. From the parameters set elements of the diagonal matrix $\hat{N}$ are as follows: ${\hat{N}}_{1}=273.8$ kg, and ${\hat{N}}_{2}=273.8$ kg, ${\hat{N}}_{3}=25.97$ kg·m^2^. Other non-zero linear and quadratic coefficients used are: $X_{r}=10,X_{|u|r}=5$, $Y_{r}=10,Y_{|v|r}=5$, $N_{u}=5,N_{|r|u}=0.5$, $N_{v}=5,N_{|r|v}=0.5$.

Based on the vehicle specifications [43,44], it was assumed that the force and torque, as well as the surge velocity, were limited. The forward force can be calculated as $\tau_{u}=T_{1}+T_{2}$ (because maximum values of forces from engines are $T_{1}=T_{2}=100$ N) and torques $\tau_{r}=T_{1}l_{2}-T_{2}l_{2}$, where $l_{2}=0.410$ m. In the tests, the maximum values of propulsion force and torque were as follows: $|\tau_{u}|\leq 120$ N and $|\tau_{r}|\leq 32$ N·m. The maximum value of surge speed is $u_{max}=1.5$ m/s (close to 3 knots). In continuous mode, the vehicle can travel for eight hours at a speed of 1 knot.

**Equipment**. The C-Ranger vehicle is used as a platform for testing algorithms and verifying them in marine research [43,44]. The control hardware system is composed of a decision-making and navigation subsystem, a CAN bus, a perception subsystem, and an actuator subsystem. Each of these subsystems has a significant impact on the vehicle’s operation. In particular, the perception subsystem contains both fundamental sensors and additional so-called new ones. The following variables can be measured: polar coordinate (Super SeaKing DST imaging sonar, Gemini 720i multibeam imaging sonar), XYZ position (XW-GPS1000 GPS), velocity (NavQuest 600 DVL-Doppler Velocity Log), attitude (Honeywell HMR3000 digital compass, Innalabs^®^ AHRS M2), angular velocity (VG951D Gyroscope), depth (Desert-star SSP-1 300PSIG Digital Pressure Sensor), communication (UWM3000 Acoustic Modems), and colour image (Kongsberg Maritime OE14-376 (PAL) Colour Camera).

### 5.3. ROPOS Vehicle

The parameters of the ROPOS vehicle can be found, e.g., [45,46]. The ROPOS vehicle dimensions, namely length, width, and height, are 1.75, 2.6, and 1.45 m [45]. Recalling [45], the signal constraints were assumed as $|\tau_{u}|\leq 1600$ N, $|\tau_{r}|\leq 1600$ N·m (although values $\tau_{u\;max}=1800$ N and $\tau_{r\;max}=2742$ N·m are possible). According to [46], the allowable velocities are $u_{max}=1.25$ m/s, $v_{max}=0.65$ m/s. The working conditions were adopted in accordance with the technical data to be realistic.

The proposed control algorithm is intended in general for vehicle models with a symmetric inertia matrix; therefore, the tests take into account the occurrence of a situation in which the center of mass has been shifted (e.g., additional mass has been added). Thus, it was assumed that $m_{13}=m_{31}=-150$ kg·m, $m_{23}=m_{32}=250$ kg·m (*Case 1*) and $m_{23}=m_{32}=500$ kg·m (*Case 2*). This corresponds (*Case 1*) to $x_{g}=0.08$ m, $y_{g}=0.1$ m, and $Y_{\dot{r}}=-68.6$ kg·m, $X_{\dot{r}}=-76.8$ kg·m. Elements of the diagonal matrix $\hat{N}$ were as follows: ${\hat{N}}_{1}=6648$ kg, and ${\hat{N}}_{2}=11786$ kg, ${\hat{N}}_{3}=7448.3$ kg·m^2^. For *Case 2*, it was $x_{g}=0.16$ m and $Y_{\dot{r}}=-137.1$ kg·m. Elements of the diagonal matrix $\hat{N}$ were as follows: ${\hat{N}}_{1}=6648$ kg, and ${\hat{N}}_{2}=$ 11,786 kg, ${\hat{N}}_{3}=7432.4$ kg·m^2^. Moreover, nonzero drag coefficients were as follows: $X_{r}$ = $N_{u}$ = 50, $Y_{r}$ = $N_{v}$ = 100, $X_{r|u|}$ = $X_{u|r|}$ = $X_{r|r|}$ = 10, $Y_{r|v|}$ = $Y_{v|r|}$ = $Y_{r|r|}$ = 10, $N_{u|u|}$ = $N_{v|u|}$ = $N_{r|u|}$ = $N_{u|v|}$ = $N_{u|r|}$ = $N_{r|v|}$ = $N_{v|r|}$ = $N_{v|v|}$ = 10.

**Equipment**. Information about ROPOS (Remotely Operated Platform for Ocean Sciences), which is a remotely operated vehicle (ROV), can be found on the Canadian Scientific Submersible Facility website [47,48]. The vehicle, whose design has been developed since 1986, has three generations. The third generation of the vehicle is equipped with various research instruments. ROPOS has, among other things, two manipulators to perform tasks underwater. In order to perform the navigation task, ROPOS is equipped with navigation sensors that are combined using a LOKI Kalman filter. With this accurate and repeatable underwater positioning, ROPOS can safely and quickly perform difficult surveys and dives. The EIVA NaviPac Pro software package is used to share, manage, and control data from sensors and systems. Telemetry is realized by Greensea Systems, whereas imaging is carried out by two HD Cameras, six pilot and tooling cameras, a 36.3 megapixel digital still camera, and over 3700 watts of lighting. The sensors subsystem is composed of an inertial navigation system (ROVINS Nano), heading primary (ROVINS Nano (Primary) IMPACT SUBSEA ISM2D COMPASS), pitch and roll (ROVINS Nano), depth (Digiquartz 8B7000-I), altimeter (Kongsberg Simrad 1007-200 kHz), sonar (Simrad MS1171 dual-frequency 330/675 kHz Digital), a Doppler Velocity Log (Nortek Compact DVL 500), USBL (iXblue GAPS, Sonardyne Ranger, Sonardyne Fusion or Kongsberg HiPAP), sound velocity (AML-1RT with an SVT), CTD (SBE 19plusV2 CTD profiler), an RF beacon (MetOcean RF-7000A1), and a strobe beacon (MetOcean ST-400A). ROPOS also has other systems used for cable laying, data management, communications, and multibeam sonar.

The second-generation ROPOS vehicle was used for the simulation tests, the parameters of which are well known from the literature, e.g., ref. [45].

The original parameters of the vehicles C-Ranger [43] and ROPOS [45] are summarized in Table 1.

### 5.4. Assumed Work Conditions

Simulation tests were performed using Matlab/Simulink in t=80 s (linear trajectory) and t=120 s (complex trajectory), with an integration step of Δ=0.05 s and the ode14x method (only for C-Ranger, complex trajectory, and the ADSMC algorithm, the ode3 method was used for numerical reasons). For testing purposes, the software was written in Matlab/Simulink from [49,50,51], and the author’s own software was used.

The desired trajectory position profiles $p_{d}=\left[x_{r},y_{r}\right]$ were selected as follows:

(56) $$p_{d}={\left[0.2\;t,\;0.2\;t\right]}^{T},$$

(57) $$p_{d}={\left[0.2\;t,\;2\sin(0.02\;t)+0.7\cos(0.03\;t)-0.3\sin(0.1\;t)\right]}^{T},$$

with $p_{d0}={\left[-0.7,0.7\right]}^{T}$.

The disturbance functions considered for both vehicles were of the form:

(58) $$\left[\begin{array}{c} f_{u}\left(t\right)\\ f_{v}\left(t\right)\\ f_{r}\left(t\right) \end{array}\right]=\left[\begin{array}{c} 15+3\sin(0.05\;t)\;N,\\ 5\sin(0.02\;t)-1.5\cos(0.01\;t)\;N,\\ 5\sin(0.02\;t)+\cos(0.04\;t)\;N\cdot m \end{array}\right].$$

Inaccuracy of the model parameters was assumed as W = 0.2 (20%).

The set of metrics used to evaluate controller performance was as follows:

(1) Time history of important variables and position tracking errors;
(2) Mean integrated absolute error $MIA=\frac{1}{t_{f}-t_{0}}\int_{t_{0}}^{t_{f}}\left|er\left(t\right)\right|dt$ where $er=x_{e},y_{e}$ (the variables $x_{e}$, $y_{e}$ were the position errors in the body-fixed frame);
(3) Mean integrated absolute control $MIAC=\frac{1}{t_{f}-t_{0}}\int_{t_{0}}^{t_{f}}\left|\tau \left(t\right)\right|dt$;
(4) Root mean square of the tracking error $RMS=\sqrt{\frac{1}{t_{f}-t_{0}}\int_{t_{0}}^{t_{f}}{∥e(t)∥}^{2}dt}$, where $∥e(t)∥=\sqrt{{\left(x_{e}\right)}^{2}+{\left(y_{e}\right)}^{2}}$.

The time history of the selected variables was necessary to determine whether the control algorithm is working properly. The indexes were used to compare the performance of various control schemes.

**Additional information based on IQV**. If the description of dynamics includes IQV, then based on the determined variables (after velocity transformation), it is possible to gain some insight into the dynamics of the system when the control algorithm is running.

In this work, two graphs and one index were used in the simulation tests, namely, known, e.g., from [21]:

(1) Time history of kinetic energy *K*;
(2) Mean kinetic energy δK=mean(K), $K=\sum_{i=1}^{3}K_{i}=\sum_{i=1}^{3}\frac{1}{2}N_{i}\zeta_{i}^{2}$ (i=1,2,3);
(3) Time history of errors between IQV and vehicle velocity, i.e., $\Delta \zeta_{i}=\zeta_{i}-\nu_{i}$.

**Remark 3.**  *On the basis of IQV, it is also possible to determine the kinetic energy reduced by each quasi-velocity, i.e., $K_{1},K_{2},K_{3}$. The errors between each IQV and its corresponding velocity represent the deformation of that velocity due to the action of dynamic couplings. In this way, it is possible to estimate how the dynamic parameters of the system affect the velocity history.*

Additionally, the χ index was introduced, representing the share of couplings in the tested model to the maximum values of couplings for this vehicle (explained in Appendix B).

*Simulation test conditions*. The operating conditions, as well as the desired trajectories, were chosen according to Assumptions 1 ÷ 8.

*Comment*. To select controller parameters, one can apply the heuristic method described in [52], which allows us to tune these parameters to improve controller quality measured by the time history of selected signals and the assumed evaluation criteria. The advantage of this method is that it is suitable for various types of control schemes (also for underactuated vehicle models). Therefore, it is useful for comparing the performance of different control algorithms. This method was used for simulation studies.

## 6. Simulation Results for Proposed Control Scheme

The proposed IQV control scheme is called here the BS-IQV (Basic Scheme using Inertial Quasi Velocities). This subsection presents the results of simulation studies performed for two vehicle models, namely C-Ranger and ROPOS. The task was to track two trajectories: linear (56) and complex (57), discussed in Section 5.

For the ROPOS vehicle, weaker and stronger couplings were considered. Based on the χ index (Appendix B), the couplings present in each model were calculated. For the C-Ranger vehicle, χ=0.2876, which means couplings greater than 28 percent; for ROPOS (weaker couplings), χ=0.0273 (less than 3 percent), and for stronger couplings, χ=0.0425 (over 4 percent). Such values indicate that for C-Ranger, the couplings are strong, while for ROPOS, they are weak (regardless of the case considered). However, the goal is to show the effectiveness of the control schemes when increasing the distance of the center of mass from the geometric center (for ROPOS).

The BS-IQV control algorithm proposed in this paper aims to track the trajectory when the vehicle’s center of mass is shifted.

**C-Ranger**. The following sets of control parameters have been selected:

(59) $$\begin{array}{cc} \mathrm{Linear}\;\mathrm{trajectory} & \;\;g_{1}=10,\;\;g_{2}=10,\;\;k=0.8,\;\;\delta =0.05;\\ & \mu_{1}=10,\;\;\mu_{2}=10,\;\;\beta_{1}=1.0,\;\;\beta_{2}=1.0,\;\;\rho_{1}=1.0;\;\;\rho_{2}=1.0, \end{array}$$

(60) $$\begin{array}{cc} \mathrm{Complex}\;\mathrm{trajectory} & \;\;g_{1}=10,\;\;g_{2}=10,\;\;k=1.1,\;\;\delta =0.04,\\ & \mu_{1}=10,\;\;\mu_{2}=10,\;\;\beta_{1}=1.0,\;\;\beta_{2}=1.0,\;\;\rho_{1}=1.0;\;\;\rho_{2}=1.0. \end{array}$$

**Figure 2:**
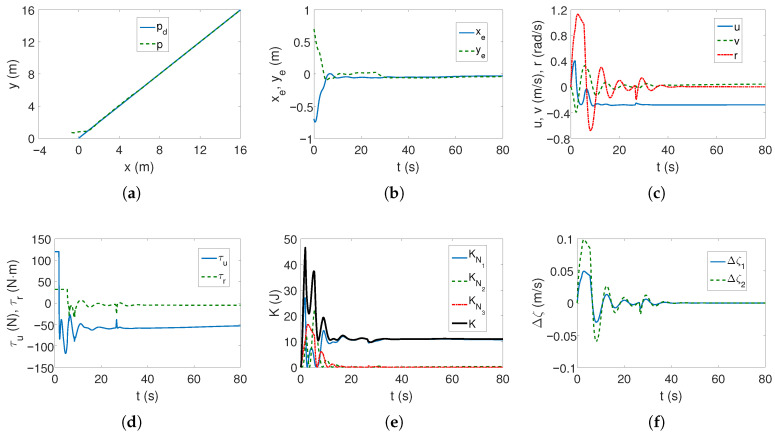
Results for C-Ranger, BS-IQV controller and linear trajectory: (**a**) desired and realized trajectory; (**b**) position errors; (**c**) velocities; (**d**) applied force and torque; (**e**) kinetic energy time history; (**f**) quasi-velocity errors $\Delta \zeta_{1},\Delta \zeta_{2}$.

As can be seen in Figure 2a,b, the desired linear trajectory is tracked, and the signal settling time is approximately 30 s. Linear velocities have small values and only the angular velocity value is higher (Figure 2c), which is caused by the inclusion of dynamics in the control algorithm. The values of the force and torque only at the beginning of the movement are maximum and then significantly decrease, as is observed in Figure 2d. The results from Figure 2e indicate that the vehicle’s movement is mainly due to surge speed, because this direction has the highest kinetic energy value. Comparing Figure 2c and Figure 2f, it can be seen that at the beginning of the vehicle’s motion, the couplings deform the speed *u* by about 1/8 of the value, and the speed *v* by even 1/4, which can be considered a significant impact (maximum error/velocity). The following mean kinetic energy values were obtained: δK=12.42 J, $\delta K_{1}=10.35$ J, $\delta K_{2}=1.01$ J, $\delta K_{3}=1.05$ J.

**Figure 3:**
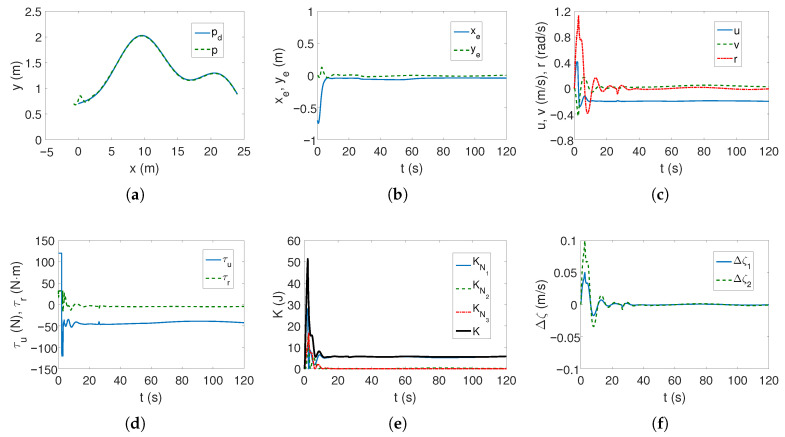
Results for C-Ranger, BS-IQV controller and complex trajectory: (**a**) desired and realized trajectory; (**b**) position errors; (**c**) velocities; (**d**) applied force and torque; (**e**) kinetic energy time history; (**f**) quasi-velocity errors $\Delta \zeta_{1},\Delta \zeta_{2}$.

The results of tracking a complex trajectory are shown in Figure 3. At the beginning, the vehicle changes position to reach the trajectory (Figure 3a,b), but as can be seen from Figure 3b, the errors in the direction of motion are slightly larger than for the linear trajectory. Also, regarding velocities, force, and torque, analogous observations can be made as before (Figure 3c,d). From Figure 3e, it can be seen that the highest kinetic energy concerns the motion at the speed *u*, and the other directions of movement do not change their values much. Similarly, Figure 3f shows that the couplings have the greatest impact on the vehicle’s movement in the transverse direction and with similar values as for the linear trajectory. Therefore, it can be concluded that the maximum coupling values existing in the vehicle model may be independent of the trajectory. The following mean kinetic energy values were obtained: δK=6.41 J, $\delta K_{1}=5.61$ J, $\delta K_{2}=0.40$ J, and $\delta K_{3}=0.39$ J.

On the basis of the obtained results, it can therefore be concluded that the proposed controller operates correctly when the center of mass is shifted by the assumed values.

**ROPOS**. The following sets of control parameters have been selected:

(61) $$\begin{array}{cc} \mathrm{Linear}/\mathrm{complex}\;\mathrm{trajectory} & \;\;g_{1}=10,\;\;g_{2}=0.7,\;\;k=0.5,\;\;\delta =0.05,\\ & \mu_{1}=10,\;\;\mu_{2}=10,\;\;\beta_{1}=10,\;\;\beta_{2}=10,\;\;\rho_{1}=1.0,\;\;\rho_{2}=1.0. \end{array}$$

Two cases were considered for this vehicle, namely weaker couplings and stronger couplings resulting from the shift of the center of mass on the longitudinal axis (*x*-axis). During the tests, it turned out that a set of parameters that was selected to provide acceptable results could be used for both linear and complex trajectories in both cases. Therefore, this proves a certain universality of the set of control parameters if it is selected correctly. Stronger couplings are understood here as larger shifts in the longitudinal axis of the *x* axis.

**Figure 4:**
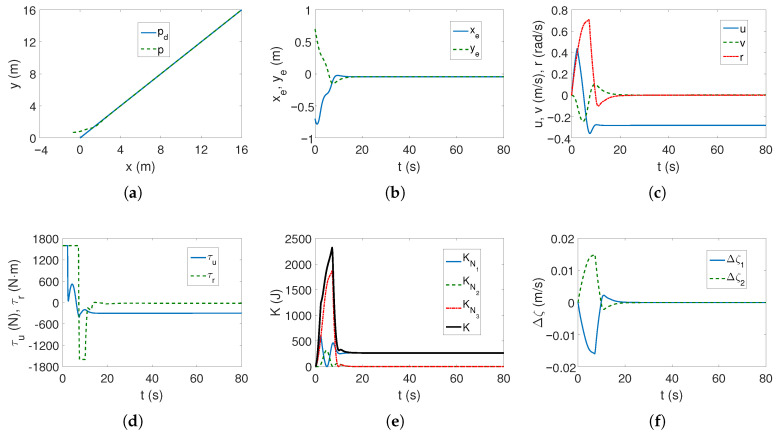
Results for ROPOS *Case 1*, BS-IQV controller and linear trajectory: (**a**) desired and realized trajectory; (**b**) position errors; (**c**) velocities; (**d**) applied force and torque; (**e**) kinetic energy time history; (**f**) quasi-velocity errors $\Delta \zeta_{1},\Delta \zeta_{2}$.

*Case 1—weaker couplings*. The linear trajectory is tracked correctly as shown in Figure 4a,b. The position errors stabilize after approximately 15 s, which is a short time considering the vehicle’s mass. These errors, however, do not tend to zero but to certain values in their neighborhood, which is consistent with the control scheme. The yaw velocity has lower values than for the C-Ranger vehicle, which can also be explained by the ROPOS vehicle mass (cf. Figure 2c and Figure 4c). The torque $\tau_{r}$ has its maximum value for about 10 s, as can be seen from Figure 4d (the dynamic parameters of the model are taken into account in the control algorithm). From Figure 4e, it is noticeable that the kinetic energy is much higher than for the C-Ranger, but this is due to the mass of the vehicle. But here, in the first phase of motion, the kinetic energy for the angular velocity *r* has the greatest value, and therefore, the vehicle rotates. Then the main part of the energy is consumed in linear motion (velocity *u*). The quasi-velocity errors shown in Figure 4f indicate that the velocity deformation is much smaller than for C-Ranger (maximum for velocity *u* about 0.04 and *v* about 0.07). This means that the dynamic couplings are also much weaker. The following mean kinetic energy values were obtained: δK=391.69 J, $\delta K_{1}=264.88$ J, $\delta K_{2}=14.16$ J, $\delta K_{3}=112.65$ J.

**Figure 5:**
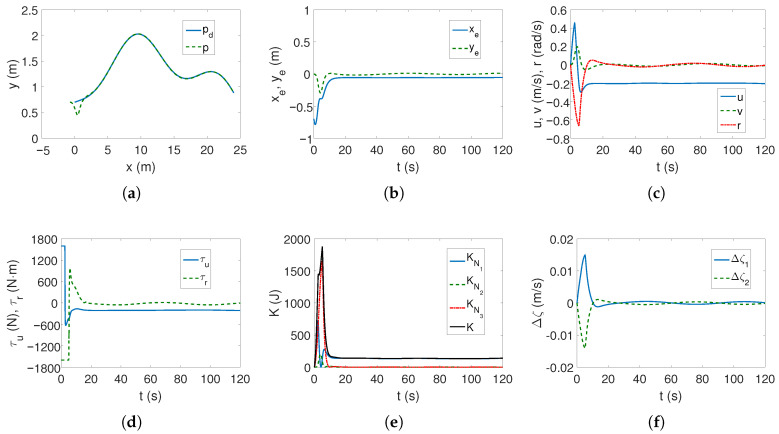
Results for ROPOS *Case 1*, BS-IQV controller and complex trajectory: (**a**) desired and realized trajectory; (**b**) position errors; (**c**) velocities; (**d**) applied force and torque; (**e**) kinetic energy time history; (**f**) quasi-velocity errors $\Delta \zeta_{1},\Delta \zeta_{2}$.

The tracking of the complex trajectory also works correctly (Figure 5a,b), but at the beginning of the movement, there is a deviation of the current trajectory resulting from taking into account the vehicle parameters in the controller. The velocities, as can be seen from Figure 5c, are low, and the thrust force and torque are maximum only when the vehicle starts moving (Figure 5d). The changes in kinetic energy compared to those calculated for the linear trajectory (Figure 5e) are not large, nor are the quasi-velocity errors (Figure 5f). This is not an unexpected result because the velocity deformation depends on the model parameters, and these have not changed. The observed differences result from the shape of the trajectory. The following mean kinetic energy values were obtained: δK=196.88 J, $\delta K_{1}=143.60$ J, $\delta K_{2}=5.05$ J, $\delta K_{3}=48.23$ J.

**Figure 6:**
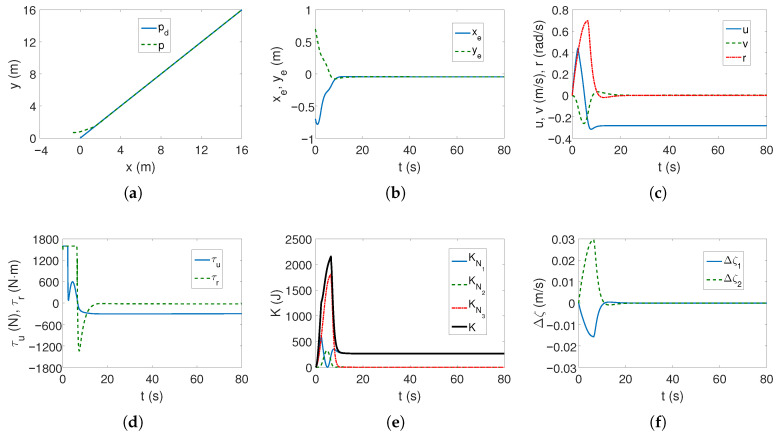
Results for ROPOS *Case 2*, BS-IQV controller and linear trajectory: (**a**) desired and realized trajectory; (**b**) position errors; (**c**) velocities; (**d**) applied force and torque; (**e**) kinetic energy time history; (**f**) quasi-velocity errors $\Delta \zeta_{1},\Delta \zeta_{2}$.

*Case 2—stronger couplings*. For the linear trajectory, when the couplings are stronger, the realized trajectory is similar to *Case 1*, and the velocities have similar values (Figure 6a,c). The differences are that the stabilization of the lateral position errors $y_{e}$ is several seconds shorter, which can be seen in Figure 6b, and the maximum torque values also operate for a shorter time (Figure 6d). It can therefore be concluded that the increase in dynamic couplings (in *x* direction) not only did not worsen the controller’s performance, but actually improved it.

It is worth noting that the maximum values of kinetic energy decreased slightly (Figure 6e) despite the increase in coupling in the longitudinal direction, with a similar time history of this energy. But Figure 6f shows that in the transverse direction the maximum couplings have doubled (at the beginning of the movement, the velocity *v* is deformed almost twice as much—about 0.12). Hence, the increase in the distance along the vehicle axis also changed the velocity deformation. The following mean kinetic energy values were obtained: δK=374.45 J, $\delta K_{1}=263.45$ J, $\delta K_{2}=12.74$ J, $\delta K_{3}=98.27$ J.

**Figure 7:**
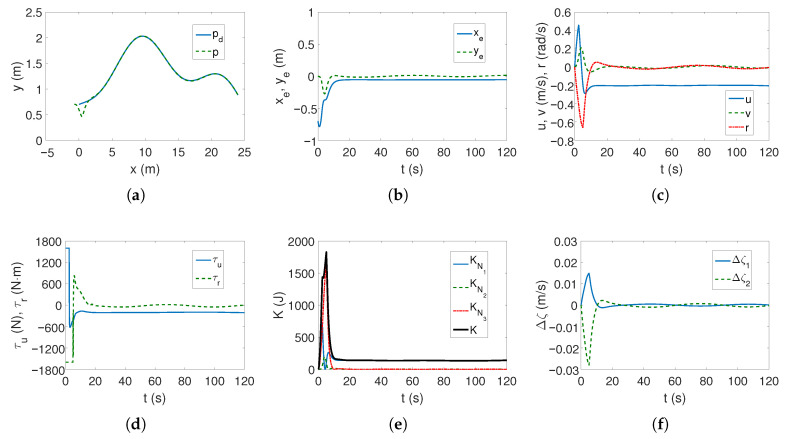
Results for ROPOS *Case 2*, BS-IQV controller and complex trajectory: (**a**) desired and realized trajectory; (**b**) position errors; (**c**) velocities; (**d**) applied force and torque; (**e**) kinetic energy time history; (**f**) quasi-velocity errors $\Delta \zeta_{1},\Delta \zeta_{2}$.

For the complex trajectory, the results shown in Figure 7a–e resemble those in *Case 1* (Figure 5a–e). There are no significant differences in the operation of the control algorithm. However, there have been changes in the time history of quasi-velocity errors because when the vehicle takes off, the couplings deform the velocity *v* by approximately 0.14 (cf. Figure 7f with Figure 5f), but this is not such a large deformation of this velocity as for C-Ranger (cf. Figure 7f with Figure 3f). The following mean kinetic energy values were obtained: δK=195.59 J, $\delta K_{1}=143.06$ J, $\delta K_{2}=4.97$ J, $\delta K_{3}=47.55$ J.

## 7. Comparison with Selected Other Control Schemes

The proposed IQV control scheme, called BS-IQV here, has been compared with two other control strategies, namely the adaptive dynamical sliding mode control (ADSMC) [4] and the global integral sliding mode control (GISMC) [5] (extended and modified in [36]). The appropriate stability proof for a three DOF vehicle moving horizontally can be found in [34]. Both control tracking algorithms were initially investigated in [17], in which better performance was obtained for them than for other tested controllers. The justification for such a comparison is also that the components of each method are backstepping and SMC. The following software was used for simulation tests: BS-IQV (modification of software [49] for three DOF and adaptation to the considered vehicles), ADSMC (software according to [50] adapted to the vehicles), and GISMC (software according to [51] adapted to the vehicles).

### 7.1. ADSMC Algorithm

The tested ADSMC control algorithm is intended, according to [4], to track the trajectory in the ideal case, or without any shift of the vehicle’s center of mass. However, here, the test concerned a situation in which the above condition is not met, and therefore, the task was to check the controller’s robustness to such a shift.

**C-Ranger**. The following set of controller parameters was selected:

(62) $$\begin{array}{cc} \mathrm{Linear}\;\mathrm{trajectory} & \;\;k_{1}=k_{2}=0.4,\;\;k_{3}=0.1,\;\;c_{1}=c_{2}=0.2,\\ & k_{s1}=k_{s2}=0.1,\;\;w_{s1}=w_{s2}=1.0, \end{array}$$

(63) $$\begin{array}{cc} \mathrm{Complex}\;\mathrm{trajectory} & \;\;k_{1}=1.0,\;\;k_{2}=0.6,\;\;k_{3}=0.2,\;\;c_{1}=c_{2}=0.5,\\ & k_{s1}=k_{s2}=0.1,\;\;w_{s1}=w_{s2}=1.0. \end{array}$$

It is worth noting that the controller parameters depend on the shape of the desired trajectory and are different for both trajectories. However, it also turned out that $k_{1},k_{2},k_{3}$, and $c_{1},c_{2}$ had the greatest impact on the performance of the control algorithm.

**Figure 8:**
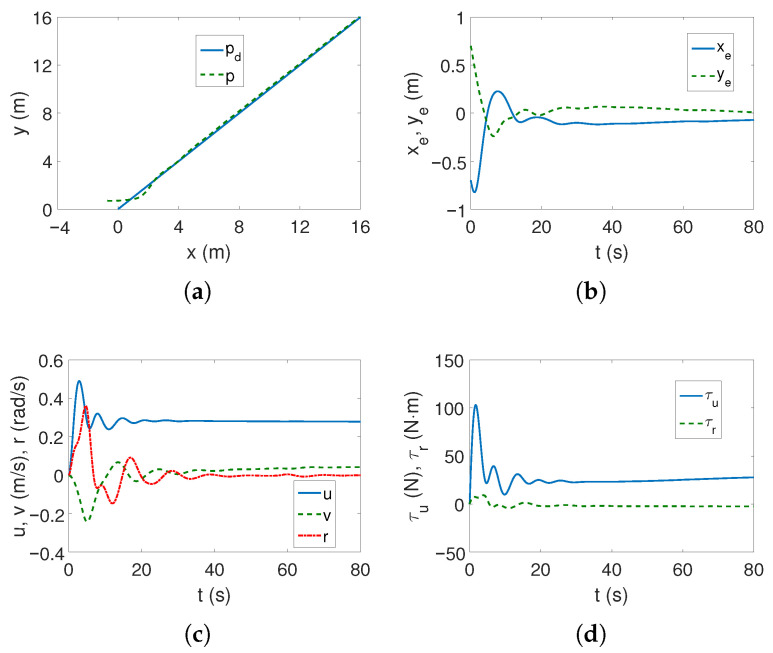
Results for C-Ranger, ADSMC controller and linear trajectory: (**a**) desired and realized trajectory; (**b**) position errors; (**c**) velocities; (**d**) applied force and torque.

For the linear trajectory, from Figure 8a,b, it can be seen that the controller is trying to cope with an unknown motion disturbance (the impact of dynamic couplings is not included in the control scheme) but the position errors do not reach a steady state within 80 s. The velocity values in Figure 8c are small, as are the force and torque values in Figure 8d, but they change for about 30 s as a result of trying to achieve the trajectory.

**Figure 9:**
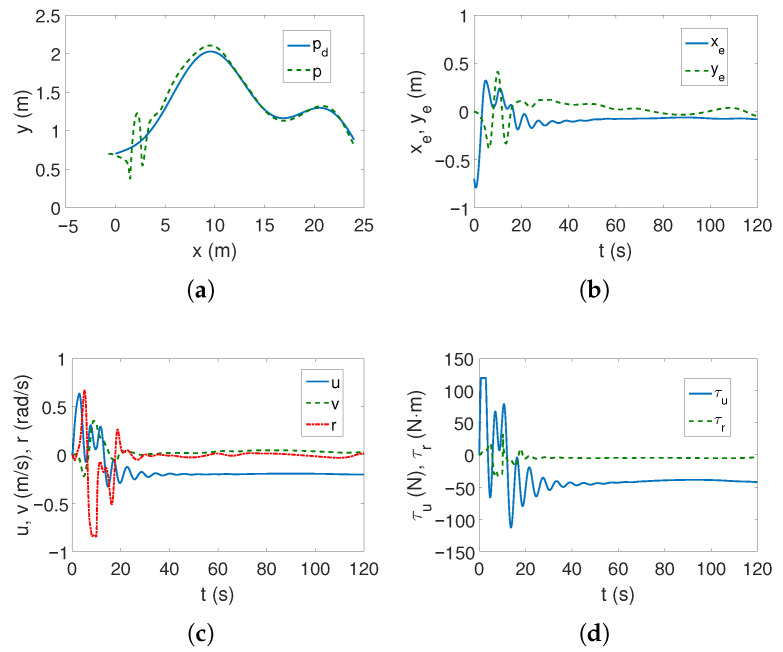
Results for C-Ranger, ADSMC controller and complex trajectory: (**a**) desired and realized trajectory; (**b**) position errors; (**c**) velocities; (**d**) applied force and torque.

In the case of the complex trajectory, the results are worse because at the beginning of the movement, the vehicle essentially changes its position (Figure 9a), whereas Figure 9b shows that the position errors are variable. In addition, as can be observed in Figure 9c, the speed values oscillate as the vehicle moves, which also applies to the force and torque shown in Figure 9d.

Based on the presented tests, it can be concluded that for the C-Ranger vehicle and the given operating conditions, the ADSMC algorithm does not provide fully acceptable results, although it tries to deal with the unexpected disturbance.

**ROPOS**. The same two cases of shifting the vehicle’s center of mass were also assumed for this algorithm.

*Case 1—weaker couplings*. The following set of controller parameters was selected:

(64) $$\begin{array}{cc} \mathrm{Linear}\;\mathrm{trajectory} & \;\;k_{1}=k_{2}=0.5,\;\;k_{3}=0.15,\;\;c_{1}=1.0,\;\;c_{2}=0.5,\\ & k_{s1}=k_{s2}=0.1,\;\;w_{s1}=w_{s2}=1.0, \end{array}$$

(65) $$\begin{array}{cc} \mathrm{Complex}\;\mathrm{trajectory} & \;\;k_{1}=k_{2}=0.3,\;\;k_{3}=1.0,\;\;c_{1}=1.0,\;\;c_{2}=0.5,\\ & k_{s1}=k_{s2}=0.1,\;\;w_{s1}=w_{s2}=1.0. \end{array}$$

When changing the vehicle, it turned out that it is worth noting that the controller parameter values should be different depending on both the model and the shape of the trajectory. Here, the change also concerned $k_{1},k_{2},k_{3}$, and $c_{1},c_{2}$.

**Figure 10:**
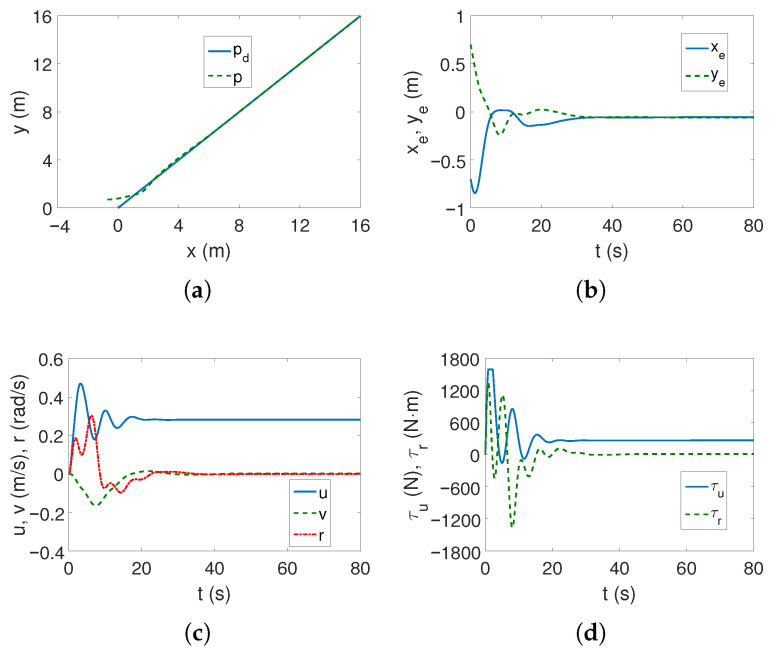
Results for ROPOS *Case 1*, ADSMC controller and linear trajectory: (**a**) desired and realized trajectory; (**b**) position errors; (**c**) velocities; (**d**) applied force and torque.

Figure 10a,b show that the algorithm fulfills its task of tracking the linear trajectory. The convergence of position errors stabilizes, which is a better result than for the C-Ranger vehicle. The speeds are low and therefore the motion is slow (well below the permissible values), as it can be noticed from Figure 10c. The force and torque have high values only when the vehicle starts to move (Figure 10d).

**Figure 11:**
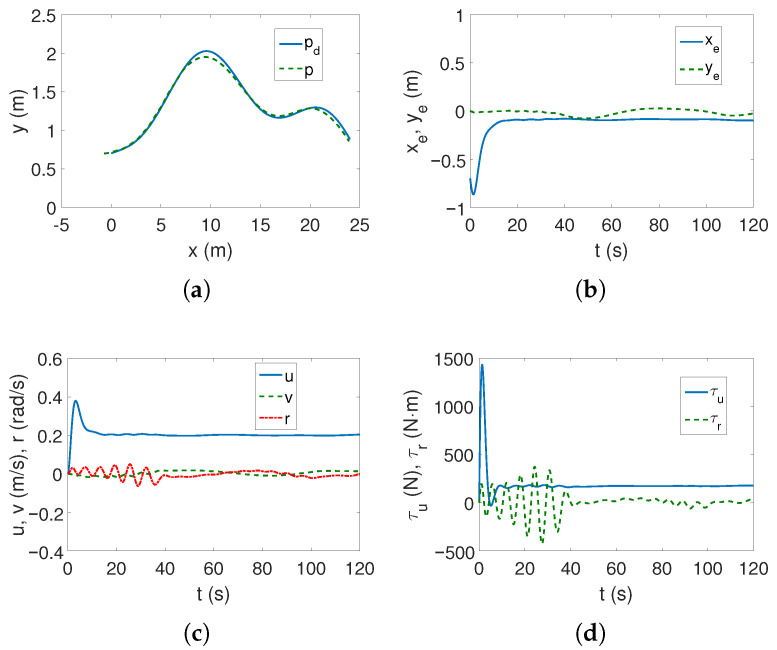
Results for ROPOS *Case 1*, ADSMC controller and complex trajectory: (**a**) desired and realized trajectory; (**b**) position errors; (**c**) velocities; (**d**) applied force and torque.

For the complex trajectory, however, the results are significantly different. The realized trajectory is not very close to the set one, which can be observed in Figure 11a, but the lateral errors $y_{e}$ change during the motion as shown in Figure 11b. Although the speeds are low (Figure 11c), the torque values oscillate, especially in the first phase of movement, as can be seen from Figure 11d.

*Case 2—stronger couplings*. The following set of controller parameters was selected:

(66) $$\begin{array}{cc} \mathrm{Linear}\;\mathrm{trajectory} & \;\;k_{1}=k_{2}=0.5,\;\;k_{3}=0.15,\;\;c_{1}=1.0,\;\;c_{2}=0.5,\\ & k_{s1}=k_{s2}=0.1,\;\;w_{s1}=w_{s2}=1.0, \end{array}$$

(67) $$\begin{array}{cc} \mathrm{Complex}\;\mathrm{trajectory} & \;\;k_{1}=0.2,\;\;k_{2}=3.0,\;\;k_{3}=0.1,\;\;c_{1}=1.0,\;\;c_{2}=0.5,\\ & k_{s1}=k_{s2}=0.1,\;\;w_{s1}=w_{s2}=1.0. \end{array}$$

After moving the center of mass further relative to the geometric center, the same control parameters as *Case 1* could be used to track the linear trajectory. But using a complex trajectory required changing $k_{1},k_{2},k_{3}$ to improve controller performance. After applying these sets of gains, the results obtained were not noticeably different from those for Case 1.

The plots for *Case 2* were omitted because when comparing Figure 10 with the results for *Case 2*, it was difficult to notice any significant differences. It can be concluded that the shift, even by a double amount in the longitudinal direction (in the *x* axis), did not affect the system time response of the examined quantities. This is important information that will be commented on in the discussion of the results.

**Figure 12:**
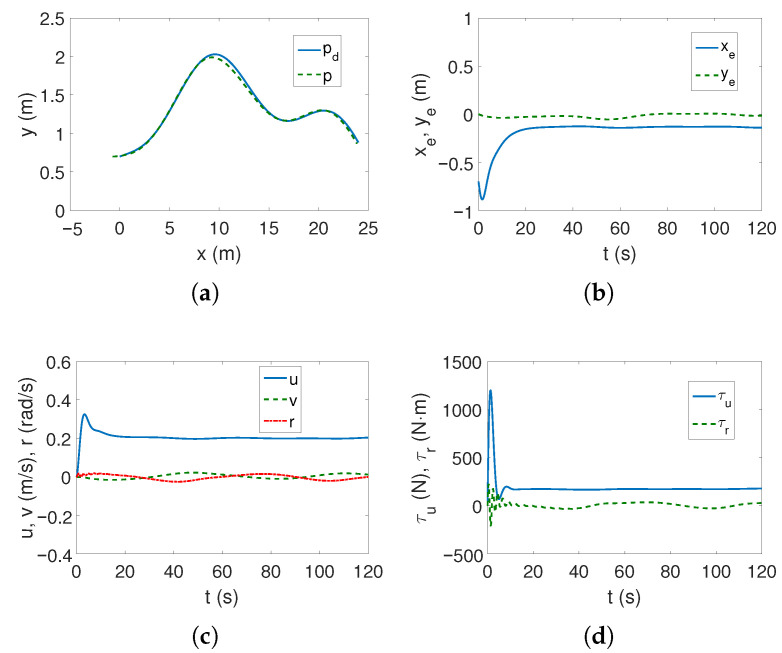
Results for ROPOS *Case 2*, ADSMC controller and complex trajectory: (**a**) desired and realized trajectory; (**b**) position errors; (**c**) velocities; (**d**) applied force and torque.

Figure 12a shows that when approaching the desired trajectory, its tracking deteriorates, and Figure 12b shows that it is clear that this error occurs in the lateral direction (*y*). The vehicle velocities are low (Figure 12c), as are the force and torque values (Figure 12d), but compared to *Case 1* (Figure 11c,d), the oscillations are smaller.

### 7.2. GISMC Algorithm

This control scheme is also intended, according to [5,36], to track the trajectory in the ideal case, or without any shift of the vehicle’s center of mass.

**C-Ranger**. The following set of controller parameters was assumed:

(68) $$\begin{array}{cc} \mathrm{Linear}\;\mathrm{trajectory} & \;\;k_{x}=20,\;\;k_{\psi }=0.3,\;\;\lambda_{1}=\lambda_{2}=0.1,\;\;\Gamma_{1}=1.0,\;\;\Gamma_{2}=0.5\\ & \beta_{1}=\beta_{2}=0.1,\;\;W=0.2, \end{array}$$

(69) $$\begin{array}{cc} \mathrm{Complex}\;\mathrm{trajectory} & \;\;k_{x}=20,\;\;k_{\psi }=0.15,\;\;\lambda_{1}=\lambda_{2}=0.1,\;\;\Gamma_{1}=1.0,\;\;\Gamma_{2}=0.5,\\ & \beta_{1}=\beta_{2}=0.01,\;\;W=0.2. \end{array}$$

As can be seen, the controller parameter values differ depending on the desired trajectory. The changes concerned $k_{\psi },\beta_{1},\beta_{2}$.

**Figure 13:**
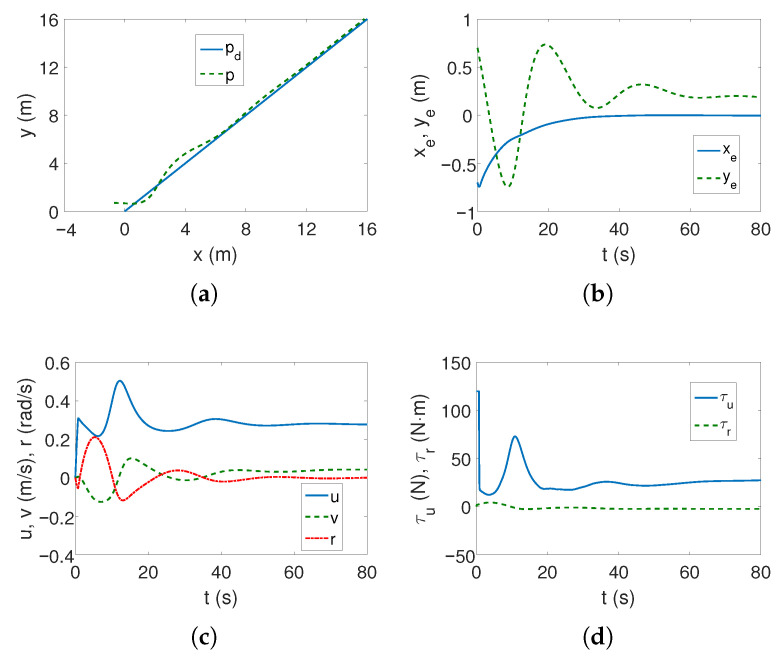
Results for C-Ranger, GISMC controller and linear trajectory: (**a**) desired and realized trajectory; (**b**) position errors; (**c**) velocities; (**d**) applied force and torque.

In the case of the linear trajectory, it turned out that it was not possible to ensure accurate tracking of the lateral variable *y* and, moreover, its value changed during the movement. This is shown in Figure 13a,b. However, the velocities are small (Figure 13c), and the force and torque are also small (Figure 13d). The lack of more accurate trajectory tracking is due to the fact that the controller is not robust to shifting the center of mass in the transverse direction.

**Figure 14:**
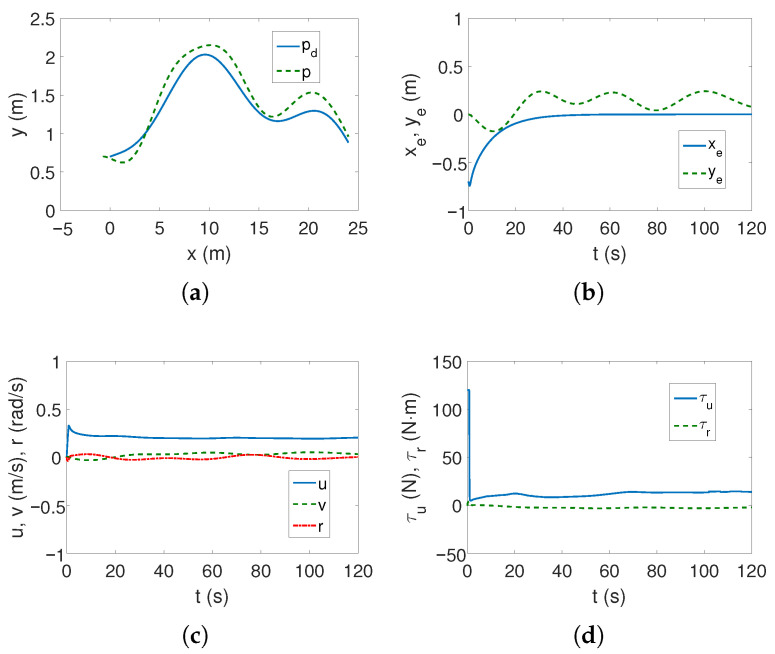
Results for C-Ranger, GISMC controller and complex trajectory: (**a**) desired and realized trajectory; (**b**) position errors; (**c**) velocities; (**d**) applied force and torque.

For a complex trajectory, the controller’s performance is also not acceptable. Figure 14a,b show that the complex trajectory is not tracked because the position errors in the lateral direction are large. This is the result of a missing component in the control scheme that would enable the task to be performed effectively. Importantly, the results from Figure 14c,d (velocities, force, and torque time responses) indicate that the algorithm is working properly.

**ROPOS**. Two cases, as previously, were considered.

*Case 1—weaker couplings*. The following set of controller parameters was assumed:

(70) $$\begin{array}{cc} \mathrm{Linear}\;\mathrm{trajectory} & \;\;k_{x}=20,\;\;k_{\psi }=1.0,\;\;\lambda_{1}=\lambda_{2}=0.1,\;\;\Gamma_{1}=1.0,\;\;\Gamma_{2}=0.5,\\ & \beta_{1}=0.5,\;\;\beta_{2}=15,\;\;W=0.2, \end{array}$$

(71) $$\begin{array}{cc} \mathrm{Complex}\;\mathrm{trajectory} & \;\;k_{x}=20,\;\;k_{\psi }=0.1,\;\;\lambda_{1}=\lambda_{2}=0.1,\;\;\Gamma_{1}=1.0,\;\;\Gamma_{2}=0.5,\\ & \beta_{1}=0.5,\;\;\beta_{2}=15,\;\;W=0.2. \end{array}$$

The controller parameter sets are almost the same for linear and complex trajectories except for $k_{\psi }$ constant.

**Figure 15:**
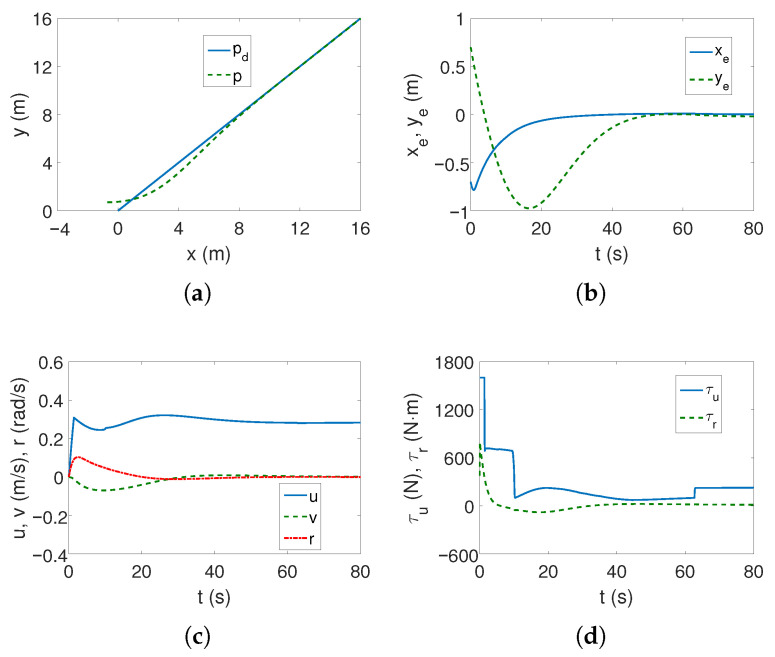
Results for ROPOS *Case 1*, GISMC controller and linear trajectory: (**a**) desired and realized trajectory; (**b**) position errors; (**c**) velocities; (**d**) applied force and torque.

Figure 15a,b show that the linear trajectory is tracked, although the time needed to establish the position errors is approximately 50 s. The vehicle moves at low speeds (Figure 15c) and the force and torque decrease quickly after it starts (Figure 15d).

**Figure 16:**
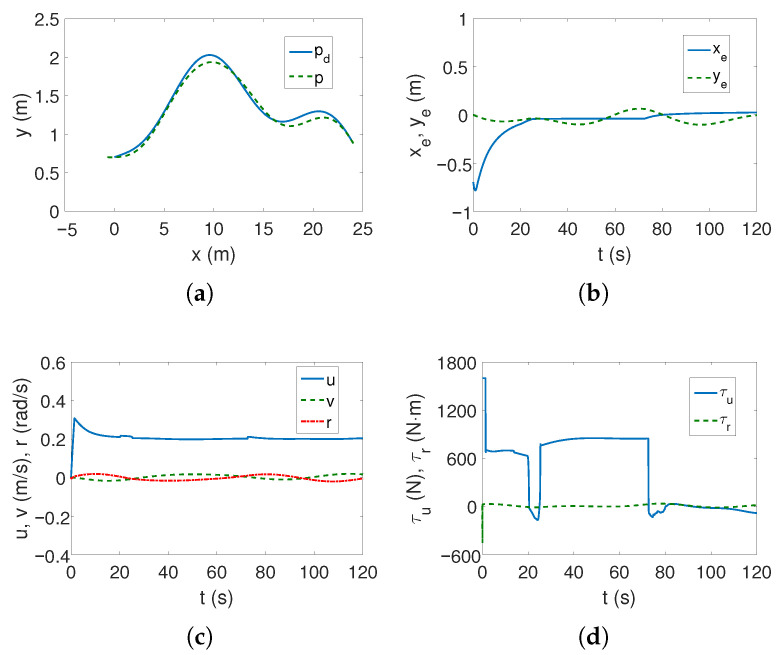
Results for ROPOS *Case 1*, GISMC controller and complex trajectory: (**a**) desired and realized trajectory; (**b**) position errors; (**c**) velocities; (**d**) applied force and torque.

For the complex trajectory, tracking the *y* variable is insufficient and the corresponding position error changes during vehicle movement but does not stabilize (Figure 16a,b). In turn, the velocity, force, and torque graphs indicate that the controller is working properly (Figure 16c,d), although it is not.

*Case 2—stronger couplings*. The same set of controller parameters, i.e., (70) and (71) was assumed.

The plots for *Case 2* were omitted because when comparing Figure 15 with the results for *Case 2* (for linear trajectory), it was difficult to notice any clear changes, which suggests that moving the center of mass even twice did not result in a loss of controller performance.

A similar observation can be made when comparing *Case 2* and Figure 16 relating to complex trajectory tracking. For the same reason, the graphs for *Case 2* were omitted. Therefore, the conclusion regarding the control algorithm is the same as before for linear trajectory tracking.

## 8. Further Analysis and Discussion of Results

### 8.1. Analysis Based on Indexes

In order to recognize the advantages and disadvantages of the tested algorithms, the index-based results should be analyzed, taking into account the time history of the variables presented previously.

Table 2 shows that for the C-Ranger vehicle tracking the linear trajectory, the smallest position errors and RMS were achieved by the proposed BS-IQV control scheme (Figure 2). Larger errors, but without convergence, were observed for the ADSMC algorithm (Figure 8). Unfortunately, the GISMC algorithm failed because the errors were too large (Figure 13). The worse performance is caused by the lack of a component compensating for the center of mass shift in the ADSMC and GISMC algorithms. However, the tracking accuracy of BS-IQV is achieved at the cost of increasing the value of input signals due to the need to take into account coupling dynamics.

Table 2 presents the results for the composite trajectory. Here, too, the BS-IQV algorithm performed the task accurately (Figure 3). Despite very small errors in the *x* axis (GISMC), this algorithm did not perform the trajectory tracking task correctly (Figure 14). The same can be said about the ADSMC controller (Figure 9). This time, however, the BS-IQV controller achieved slightly lower control effort than the ADSMC.

Based on this analysis, it can be concluded that for the C-Ranger vehicle (with a dry weight of just over 200 kg), the designed control scheme in which dynamic couplings are taken into account in the model worked best. On the contrary, the remaining algorithms that did not contain a component compensating for the shift of the center of mass did not perform the task of tracking the selected trajectories correctly. Therefore, in this case, it is difficult to say that they are suitable for tracking in the existing operating conditions and for the assumed vehicle.

Table 3 presents the criteria results for the ROPOS vehicle, linear trajectory, and tested control algorithms. Two cases were considered, i.e., C1 (weaker couplings understood as a smaller distance from the center of mass in the *x* direction and C2 (stronger couplings in the sense of further distance along the same axis). Again, it turned out that the BS-IQV algorithm tracked the trajectory with greater accuracy than the other controllers. However, taking into account dynamic couplings resulted in an increase in control effort (τ). This is often a feature of this type of algorithm. It is worth noting that the linear trajectory tracking task was also performed using the ADSMC and GISMC algorithms. The difference in performance is visible because the results for the ADSMC control scheme are slightly worse but similar to the BS-IQV results. However, for the GISMC algorithm, they are much worse, although perhaps in some situations they would be acceptable. Additionally, for the GISMC strategy, the control effort is much lower. What is interesting is the observation that the increase in the distance of the center of mass from the geometric center did not cause significant changes in the performance of any of the control algorithms (C2). It even turned out that the results obtained for C2 were slightly more favorable than for C1. This is a valuable tip because usually control strategies designed for models containing couplings take into account the shift in the *x* direction. Therefore, this could mean that by selecting the control parameters correctly (i.e., in such a way as to ensure acceptable results), one can also effectively track the desired linear trajectory using a system model with a diagonal inertia matrix (i.e., ignoring dynamic couplings between variables).

Table 4 summarizes the performance of tracking the complex desired trajectory. The obtained results indicate that although in the *x* direction the position errors using the BS-IQV algorithm were not the smallest, the errors in the *y* direction were definitely smaller than for the other control schemes, and, moreover, the RMS also had the smallest values (for C1 and C2). This is also confirmed by Figure 5 and Figure 7. An additional benefit is that the control effort of this algorithm was lower than that of the GISMC algorithm (τ). Comparing the RMS indexes for the ADSMC algorithm in cases C1 and C2, it can be seen that the RMS value deteriorated for C2. However, this is misleading because for C2 (Figure 12) the movement is more stable than for C1 (Figure 11). It is because, for C1, one-third of the way through the motion, the vehicle oscillates, trying to cope with unexpected couplings. This behavior was not observed for C2. From this, one can conclude that increasing the value of $x_{g}$ improved the tracking of the complex trajectory. It is worth noting that this algorithm still produces larger tracking errors than if the BS-IQV algorithm were used. Moreover, the GISMC algorithm, despite low RMS values, cannot stabilize the $y_{e}$ error, i.e., in the lateral direction (Figure 16). Therefore, relying solely on indexes may be misleading. Additionally, greater control effort is required than when using the BS-IQV or the ADSMC control strategy.

Comparing the results from the tables and the time histories of selected variables, it turned out that relying solely on indexes provides information that may be misleading and does not give true information about the operation of the control algorithms. Therefore, it is necessary to analyze the graphs obtained in simulations.

### 8.2. Discussion

The results obtained can be summarized as follows.

1. If there is a shift in the vehicle’s center of mass, it is worth using a control scheme dedicated to the trajectory tracking task because it includes components to compensate for the disturbances resulting from such a situation (here, the BS-IQV controller).
2. With the assumptions adopted for the tests (i.e., slow motion of the vehicle about 0.5 m/s or below 1 m/s in the steady state phase of motion), slowly changing linear and complex trajectories, shift of the center of mass by several cm in the longitudinal and lateral directions x,y up to a dozen or so in the longitudinal direction *x*) it was shown that the ADSMC algorithm provided acceptable results for the ROPOS vehicle, linear trajectory C1, C2, and complex C2. The GISMC algorithm also worked correctly for the ROPOS vehicle when tracking the linear trajectory of C1, C2 (although with a large position error). For the C-Ranger vehicle, the performance was much worse.
3. It turned out, based on the simulations performed, that for the high-mass ROPOS vehicle, the ADSMC and GISMC algorithms were more robust to the occurrence of dynamic couplings than for the lower-mass vehicle (over ten times) C-Ranger. However, this is related to the value of the χ index, which indicates that in the case of C-Ranger, the dynamic couplings are much stronger than in the case of ROPOS.
4. A control algorithm that does not take into account couplings—when these couplings arise, the phenomenon of oscillatory movements may occur (the ADSMC control scheme, Figure 11), which shows that the controller is trying to cope with the situation. Moreover, in the lateral direction (less robust to displacement), position errors change with oscillations during movement (in the ADSMC algorithm, Figure 9, Figure 11 and Figure 12, and in the GISMC algorithm, Figure 13, Figure 14 and Figure 16).
5. The largest position errors for the ADSMC controller were in the longitudinal *x* direction, while for the GISMC controller, they were in the *x* or *y* direction, depending on the vehicle and the tracked trajectory. Therefore, it is impossible to say which direction is more sensitive to the shift of the center of mass when the GISMC algorithm is running.

If a control strategy is designed assuming that dynamic controls are ignored, it should be checked for situations in which these couplings will occur (due to the shift of the center of mass). This applies to tests completed with simulation verification. This is basically a necessity for two reasons.

1. The situation in which a diagonal inertia matrix is assumed is ideal and, therefore, from a practical point of view, of little use (unless the control method has been experimentally verified for a real vehicle, because then the operating conditions for which the experiment was performed were given).
2. Information about robustness to the presence of mass shift is a measure of the usefulness of the proposed strategy, and there are some that, in certain situations and for certain trajectories, can be used with a small shift of the center of mass, while others will not cope when such a phenomenon occurs. Therefore, the research should determine the maximum values of the displacement of this center, the operating conditions for which the algorithm is useful for tracking, e.g., trajectory, dynamic parameters of the vehicle, movement velocity, and maximum thrust forces.

**Summary**. Evaluation of the results of the tests performed is given in Table 5. Explanation of used symbols: LT—linear trajectory, CT—complex trajectory, P.Error—position error, MCSR—mass center shift robustness, CIV—control input value, CEBV—control efficiency for both vehicles, ‘+’—acceptable results, ‘−/+’—sometimes acceptable results, ‘−’—unacceptable results, ‘++’—better results (smaller values of control signals), No*—control scheme effective only for ROPOS and linear trajectory.

Table 5 shows that the proposed BS-IQV control scheme (dynamics model with symmetric inertia matrix) proved effective in all tests. However, the position errors for the ADMSMC algorithm were only sometimes acceptable, and for the GISMC, none of them were. It can also be seen that the use of the BS-IQV controller leads to acceptable errors of the uncontrollable variable *y*, but the use of the other algorithms (dynamics model with diagonal inertia matrix) leads to high values of these errors. Moreover, it was found that only BS-IQV is robust to the center of mass shift, while the ADMSMC and GISMC algorithms do not show such robustness, which is manifested by the increase in error values. To perform the trajectory tracking task, higher values of the control force and torque are required for the BS-IQV control scheme than when using the other algorithms. However, lower values of these variables are irrelevant when the control task is not properly performed. The proposed algorithm proved to be effective for both vehicle models and for both trajectories. The ADMSMC and GISMC control schemes proved to be effective only exceptionally. It is worth noting that the tests assumed real limitations of the drive motors so that realistic results could also be obtained. Sometimes in the literature, there is a lack of such limitations, and then it is possible to obtain effective operation of the control algorithm. However, such an approach, from a technical point of view, is unacceptable because in reality, such a vehicle does not exist, although the simulation results give the impression that the trajectory tracking task can be realized.

*Conclusion from the tests*. Sometimes it is possible to select the operating conditions of the control algorithm or not to impose restrictions on the control signals, which will lead to the correct tracking of the desired trajectory (when a diagonal inertia matrix is assumed, i.e., when dynamic couplings are omitted). However, verification of such an algorithm for a different vehicle model or in different operating conditions will reveal its weaknesses. The problem of robustness to the shift of the center of mass is therefore important and should be taken into account when designing new control strategies. As tests have shown, the use of the ADMSMC and GISMC control strategies has only exceptionally produced acceptable results.

## 9. Conclusions

This work proposes a motion control scheme for a vehicle moving horizontally, taking into account robustness to imperfectly known model parameters, environmental disturbances and shifts of its center of mass. The focus was on the issue of shifting the center of mass in relation to the geometric center of the vehicle. It has been shown (in simulation) that the control method developed for the model containing such a shift ensures acceptable performance regarding position errors under the assumed operating conditions, including realistic limits on the driving forces and the vehicle speed. The performance of the designed controller was compared with the results obtained for other algorithms that were dedicated to controlling vehicles with a diagonal inertia matrix model (omitting dynamic couplings). For two such control schemes, the work conditions for which trajectory tracking can be achieved with acceptable accuracy regarding position errors are indicated.

Despite the fact that the most accurate tracking of the trajectory position, taking into account the mass displacement, was obtained for the control scheme intended for this purpose, it cannot be said that the existing strategies proposed for the model without couplings are useless. On the contrary, there are operating conditions that allow the successful implementation of the trajectory tracking task. This is also where they should focus on finding solutions to this problem.

Since control schemes for models with a diagonal inertia matrix are very often proposed in the literature, it seems necessary to check the robustness of these schemes to the possibility of displacing the center of mass. It is essential to define under what conditions they will operate effectively. Checking operation in ideal conditions obviously makes sense—it will not be an experimental verification, for which the operating conditions of the controller are also given, but it gives some insight into the behavior of the model-controller system. However, verification of the algorithm only through simulation, without information about the vehicle’s motion when the center of mass is moved, is insufficient because it may be impractical, and the controller itself may not be very robust to disturbances resulting from such a situation.

From the presented simulation results, it can be concluded that the control algorithms are robust to shift changes in the longitudinal direction (such a test was performed for the ROPOS vehicle). It is worth noting that the control schemes designed for models with couplings concern shifts in this direction.

Future research should focus on identifying such operating conditions for models with a diagonal matrix of inertia that will allow the practical application of these algorithms based on simulation studies. Currently, this problem is often ignored or solved to a narrow extent.

## Figures and Tables

**Table 1:** Parameters of C-Ranger [43] and ROPOS [45].

	C-Ranger	ROPOS	
Symbol	Value	Value	**Unit**
*m*	206	2268	kg
$m_{11}$	273.8	6648	kg
$m_{22}$	273.8	11,786	kg
$m_{33}$	28.6	7457	kg·m^2^
$X_{u}$	120	725	kg/s
$Y_{v}$	90	1240	kg/s
$N_{r}$	18	1804	kg·m^2^/s
$X_{u|u|}$	90	1000	kg/m
$Y_{v|v|}$	90	525	kg/m
$N_{r|r|}$	15	72	kg·m^2^

**Table 2:** Performance for C-Ranger.

		Linear			Complex	
Index	BS-IQV	ADSMC	GISMC	BS-IQV	ADSMC	GISMC
MIA $x_{e}$	0.0672	0.1217	0.0858	0.0609	0.1030	0.0588
MIA $y_{e}$	0.0587	0.0619	0.3046	0.0092	0.0652	0.1481
RMS ||e||	0.1607	0.2033	0.3634	0.1046	0.1715	0.1621
MIAC $\tau_{u}$	59.611	26.860	26.805	44.490	44.941	12.335
MIAC $\tau_{r}$	7.1325	2.4080	2.1623	5.2457	5.9435	2.3625
MIAC ∑τ	66.744	29.268	28.967	49.746	50.885	14.698

**Table 3:** Performance for ROPOS and linear trajectory.

	BS-IQV	BS-IQV	ADSMC	ADSMC	GISMC	GISMC
Index	(C1)	(C2)	(C1)	(C2)	(C1)	(C2)
MIA $x_{e}$	0.0821	0.0815	0.0981	0.0983	0.0853	0.0853
MIA $y_{e}$	0.0677	0.0636	0.0697	0.0694	0.2964	0.2983
RMS ||e||	0.1906	0.1861	0.2048	0.2046	0.4913	0.4939
MIAC $\tau_{u}$	335.06	338.07	315.91	316.18	238.66	238.35
MIAC $\tau_{r}$	229.26	193.77	123.38	123.24	41.677	40.990
MIAC ∑τ	565.32	531.84	439.29	439.42	280.34	279.34

**Table 4:** Performance for ROPOS and complex trajectory.

	BS-IQV	BS-IQV	ADSMC	ADSMC	GISMC	GISMC
Index	(C1)	(C2)	(C1)	(C2)	(C1)	(C2)
MIA $x_{e}$	0.0861	0.0854	0.1207	0.1776	0.0792	0.0792
MIA $y_{e}$	0.0157	0.0153	0.0253	0.0206	0.0559	0.0566
RMS ||e||	0.1490	0.1467	0.1779	0.2266	0.1722	0.1725
MIAC $\tau_{u}$	239.40	239.48	190.91	190.05	474.18	474.61
MIAC $\tau_{r}$	123.10	120.85	69.146	24.147	11.500	11.514
MIAC ∑τ	362.50	360.33	260.06	214.20	485.68	486.12

**Table 5:** Evaluation of control schemes.

	BS-IQV	BS-IQV	ADSMC	ADSMC	GISMC	GISMC
	LT	CT	LT	CT	LT	CT
P.Error	+	+	−/+	−/+	−	−
Error $y_{e}$	+	+	−	−	−	−
MCSR	+	+	−	−	−	−
CIV	+	+	++	++	++	++
CEBV	Yes	Yes	No*	No	No*	No

## Data Availability

The data presented in this study are contained in the article itself.

## Appendix A

Matrix element values from (3):

(A1) $$m_{11}=m-X_{\dot{u}},\;\;m_{13}=m_{31}=-my_{g}-X_{\dot{r}},\;\;m_{22}=m-Y_{\dot{v}},$$

(A2) $$m_{23}=m_{32}=mx_{g}-Y_{\dot{r}},\;\;m_{33}=J_{z}-N_{\dot{r}},$$

(A3) $$c_{13}=-m_{22}v-m_{23}r,\;\;c_{23}=m_{11}u-m_{13}r,$$

(A4) $$d_{11}=X_{u}+X_{|u|u}\left|u\right|+X_{|r|u}|r|,\;\;d_{13}=X_{r}+X_{|r|r}\left|r\right|+X_{|u|r}\left|u\right|,$$

(A5) $$d_{22}=Y_{v}+Y_{|v|v}\left|v\right|+Y_{|r|v}|r|,\;\;d_{23}=Y_{r}+Y_{|v|r}\left|v\right|+Y_{|r|r}\left|r\right|,$$

(A6) $$d_{31}=N_{u}+N_{|u|u}\left|u\right|+N_{|v|u}\left|v\right|+N_{|r|u}|r|,\;\;d_{32}=N_{v}+N_{|v|v}\left|v\right|+N_{|r|v}\left|r\right|+N_{|u|v}\left|u\right|,$$

(A7) $$d_{33}=N_{r}+N_{|v|r}\left|v\right|+N_{|r|r}\left|r\right|+N_{|u|r}\left|u\right|.$$

The water resistance is defined by (A4)–(A7), which is composed of linear and quadratic drag.

## Appendix B

The proposed index concerns the share of couplings in the vehicle model and is related to the maximum couplings theoretically possible to obtain for the model under consideration. The matrix $\left|\right|\hat{\Phi }\left|\right|$ from (5) is of key importance in the proposed method of assessing couplings.

The index of dynamic couplings impact is defined as:

(A8) $$\chi =\frac{\left|\right|\hat{\Phi }\left|\right|-1}{\left|\right|{\hat{\Phi }}_{max}\left|\right|-1},$$

where $\left|\right|\hat{\Phi }\left|\right|$ is the matrix norm for the tested vehicle model while $\left|\right|{\hat{\Phi }}_{max}\left|\right|$ is the norm of the model matrix assuming possible maximum couplings (from theoretical calculations).

According to *Remark 1*, ${\hat{N}}_{3}$ is determined instead of $N_{3}$ because the nominal elements of the matrix $\hat{N}$ are known. It is assumed that maximum couplings will occur when ${\hat{N}}_{3}=0$ in ${\hat{N}}_{3}={\hat{m}}_{33}-\left({\hat{m}}_{13}/{\hat{m}}_{11}\right)-\left({\hat{m}}_{23}/{\hat{m}}_{22}\right)$ (the minimum value of the third element of the matrix $\hat{N}$). This situation will occur, for example, if $0.5\;{\hat{m}}_{33}={\hat{m}}_{13}^{2}/{\hat{m}}_{11}$ and $0.5\;{\hat{m}}_{33}={\hat{m}}_{23}^{2}/{\hat{m}}_{22}$. Then the maximum values of ${\hat{m}}_{13\;max},{\hat{m}}_{23\;max}$ can be determined as follows ${\hat{m}}_{13\;max}=\sqrt{0.5\;{\hat{m}}_{33}\;{\hat{m}}_{11}}$ and ${\hat{m}}_{23\;max}=\sqrt{0.5\;{\hat{m}}_{33}\;{\hat{m}}_{22}}$. These values are substituted into matrix $\left|\right|{\hat{\Phi }}_{max}\left|\right|$, while the values for the tested model are substituted into matrix $\left|\right|\hat{\Phi }\left|\right|$.

Interpretation of this index is that the higher its value, the greater the dynamic couplings in the model. If the value of χ is several percent, the coupling can be considered weak and with such a model, control is easier, although it does not necessarily lead to acceptable results. Index values of higher than 20 percent show that the couplings are so strong that they can significantly make control difficult or to make impossible correct trajectory tracking.
